# Microwave-Assisted Conversion of Low-Rank Lignite into Hierarchical Activated Carbon: Molecular Insights into Efficient Post-Combustion CO_2_ Capture

**DOI:** 10.3390/ijms27146123

**Published:** 2026-07-08

**Authors:** Anusorn Boonpoke, Sirasit Meesiri, Saksit Imman, Boonyawan Yoosuk, Wajussakorn Kanjana, Surachai Wongcharee

**Affiliations:** 1 School of Energy and Environment, University of Phayao, Phayao 56000, Thailand; iamanusorn@gmail.com (A.B.); sirasit.khing11@gmail.com (S.M.); saksit.im@up.ac.th (S.I.); 2 Atmospheric Pollution and Climate Change Unit (APCC), School of Energy and Environment, University of Phayao, Phayao 56000, Thailand; 3 Clean Fuel Technology and Advanced Chemistry Research Team, National Energy Technology Center, National Science and Technology Development Agency, Pathum Thani 12120, Thailand; boonyawan.yoo@entec.or.th; 4 Faculty of Engineering, Mahasarakham University, Khamriang, Maha Sarakham 44150, Thailand; wajussakorn.k@msu.ac.th

**Keywords:** activated carbon, microwave-assisted, chemical activation, CO_2_ capture

## Abstract

Lignite-derived activated carbon (L-AC) was fabricated via a microwave-assisted KOH activation process using a low-rank Mae Moh lignite and explored its potential as an adsorbent solid for post-combustion CO_2_ capture. Optimization of the KOH ratio, microwave irradiation power, and activation time gave rise to a product with a BET surface area of 1349 m^2^ g^−1^ and total pore volume of 0.78 cm^3^ g^−1^, which represented 165 times and 78 times enhancement compared with that of the initial lignite, respectively. Scanning electron microscope (SEM) images proved the formation of a hierarchical macropore–mesopore–micropore structure, whereas Raman (I_D_/I_G_ = 1.83) and Fourier-transform infrared spectroscopy analyses revealed a graphitic-like structure rich in defects with the existence of C=O and C–O–C functional groups involved in the Lewis acid–base interaction between L-AC and CO_2_ molecules. Dynamic fixed-bed breakthrough tests performed at temperatures of 298, 328, and 353 K under post-combustion relevant conditions (CO_2_ concentration: 15%, pressure: 1 atm) yielded CO_2_ equilibrium uptake capacities of 47.34, 34.37, and 21.34 mg g^−1^, respectively, with outstanding cyclic stability achieved after six consecutive adsorption–desorption cycles of temperature swing adsorption–desorption at 393 K. Among the seven nonlinear kinetic models, the Avrami, FL-PFO, and general-order models exhibited the highest fitting accuracy (R^2^ = 0.9994–0.9998), suggesting that CO_2_ adsorption onto L-AC proceeds through heterogeneous, multi-stage adsorption kinetics. A Weber–Morris intra-particle diffusion analysis identified a three-stage sequential transport mechanism in which mesopore diffusion constitutes the primary rate-limiting step. Thermodynamic parameters confirmed spontaneous (ΔG° = −24.20 to −26.87 kJ mol^−1^), exothermic (ΔH° = −9.42 kJ mol^−1^), and entropy-assisted adsorption (ΔS° = +49.93 J mol^−1^ K^−1^) consistent with a physisorption mechanism, corroborated by a low activation energy of 9.11 kJ mol^−1^. These findings demonstrate the viability of low-rank lignite as a low-cost precursor for the scalable synthesis of high-performance carbonaceous CO_2_ adsorbents for post-combustion capture applications.

## 1. Introduction

The progressive accumulation of carbon dioxide (CO_2_) in the atmosphere, driven primarily by the combustion of fossil fuels for power generation, remains one of the most pressing environmental challenges of the twenty-first century [1,2]. In 2024, the hottest year on record global temperatures exceeded pre-industrial levels by more than 1.5 °C for the first time, and atmospheric CO_2_ concentrations exceeded pre-industrial levels by more than 52%, further intensifying international efforts to advance carbon capture technologies [3]. Coal-fired power stations rank amongst the most significant stationary sources of CO_2_ emissions in the world, emitting flue gases with the CO_2_ concentration range of 12–15 vol% [2,4]. Against this backdrop, the use of carbon capture, utilization, and storage (CCUS) technology can be viewed as crucial technology that is able to considerably reduce the emissions of CO_2_ produced by fossil fuel power stations, as well as industry, such as cement, steel, and petrochemical industries [5]. Interest in the scientific community in CCUS technologies increased in 2024, and the number of scientific publications rose by 11.4% compared to 2023. Currently, there are many post-combustion technologies of CO_2_ capture, but the best industrial technology is aqueous amine scrubbing, mostly using monoethanolamides (MEAs); nevertheless, the process requires energy expenditure of 6.1–14.5 GJ t^−1^ CO_2_ due to solvent regeneration, compression, and transportation [6]. Moreover, the process of using amine-based solvents has other problems, such as the volatility of amines, reactor corrosion, and significant energy expenditure on regenerating solutions. Thus, considerable research is being conducted today in the field of developing solid adsorbents, which include activated carbon, zeolites, MOF, COFs, and amine-containing solids [7].

The utilization of solid adsorbents, such as zeolites, metal organic frameworks (MOFs), covalent organic frameworks (COFs), silica-supported amines, and carbonaceous adsorbents, has various benefits compared to liquid scrubbing technology, ranging from low regeneration energy demand to low corrosivity of equipment, high thermal and chemical stability, and easier handling [7,8,9,10]. However, zeolites have relatively high sensitivity to moisture and experience significant dealumination when subjected to real-world flue gas composition, whereas MOFs with high surface areas (1500–10,000 m^2^ g^−1^) are hindered by their prohibitively expensive preparation process, high susceptibility to moisture, and poor mechanical stability [8,11]. Among all solid adsorbents, activated carbons (ACs) have garnered increasing interest because of their extremely high specific surface area (400–2000 m^2^ g^−1^), easily adjustable pore size, suitable surface chemistry, and more importantly, intrinsic hydrophobic and non-polar nature that confers excellent resistance towards the competing adsorption of water vapor [4,12,13]. Various fixed bed experiments have shown that the adsorption capacity remains constant or even rises with the presence of moisture in the flue gas compared to the significant losses (15–17%) experienced by zeolites and MOFs [11]. AC adsorbents also benefit from being less expensive than MOFs and zeolites, easy accessibility to inexpensive sources of carbonaceous materials, and commercial readiness in terms of the production process [6,14]. AC adsorbents derived from coal have shown CO_2_ adsorption capacities of 0.03–0.91 mmol g^−1^ under post-combustion flue gas conditions, accompanied by relatively high BET surface areas ranging from 661 to 1100 m^2^ g^−1^ [2,15,16,17].

The textural and adsorption properties of activated carbon are primarily determined by the choice of the initial carbonaceous precursor and the activation method used. Activated carbon is produced from carbon-rich and ash-low precursors through physical and/or chemical activation. In particular, activated carbon production through chemical activation using potassium hydroxide (KOH) is highly appreciated due to the extremely high surface area values (726–1247 m^2^ g^−1^), the formation of an intensive micropore network, and relatively high yield values (30–50 wt%) at medium activation temperatures (937–1173 K) [18,19,20]. Chemical activation with KOH is based on a series of reactions, including carbon gasification with K_2_O, Boudoir reaction, and potassium intercalation within the graphite layer of the carbon material to form a hierarchical structure comprising micropores and mesopores [21,22]. It should be noted that coal precursors provide AC with better textural properties than the materials produced using biomass-based precursors. This phenomenon can be attributed to higher amounts of fixed carbon and greater orderliness of the graphite structure [18,19,20].

One of the recently developed approaches to the synthesis of AC includes microwave-assisted heating. Unlike classical resistive heating of the precursor in an electric furnace, microwave radiation with a frequency of 2.45 GHz ensures a fast volumetric, non-contact dielectric heating of the coal–KOH system [23]. Microwave activation leads to 20% lower energy consumption per volume unit of AC produced, up to 55% increased yield [24], and a considerably shorter time for the process. Recent studies have shown that the microwave-assisted activation of coal-based precursors produces N-doped ultra-microporous activated carbon with a BET surface area larger than 1500 m^2^ g^−1^ and an extremely high CO_2_ uptake capacity at room temperature [21]. Fast volumetric heating and consequent potassium diffusion within the carbon structure provide more homogenous micropore formation, thus leading to a narrow micropore size distribution [23].

Low-rank lignite represents an underutilized yet strategically important carbonaceous precursor for AC synthesis. Lignite is among the most abundant and lowest-cost coal reserves globally, and Thailand holds substantial deposits at the Mae Moh basin in Lampang Province [25]. Despite its relatively low fixed-carbon content and high moisture and oxygen content compared to higher-rank coal, lignite’s abundant oxygen-bearing functional groups, including carboxyl, hydroxyl, and ether moieties, may be leveraged during KOH activation to generate a defect-enriched, surface-active carbon framework with enhanced affinity for CO_2_ [26]. Furthermore, the conversion of an otherwise low-value by-product from power generation into a value-added adsorbent aligns with circular economy principles and supports the sustainable management of lignite mining residues. Previous studies have explored the KOH activation of low-rank coal for dye adsorption applications [25], but a systematic investigation of lignite-derived AC for gas-phase CO_2_ capture under fixed-bed conditions, with comprehensive kinetic, thermodynamic, and mechanistic analysis, has not been reported.

Although there is a wealth of studies in the literature concerning the promising adsorption capacity of lignite-based adsorbents and the energy savings provided by microwave-assisted activation, there have been few studies focusing on the development of CO_2_ adsorbents using low-rank lignite as the raw material, KOH as the activating agent, and a microwave activation process. Especially, no investigation has reported a detailed physicochemical characterization of such activated carbon, which comprises textural analyses by BET/BJH, surface chemistry by Raman and FTIR, morphology characterization by SEM, along with its performance of fixed-bed breakthrough experiments, modeling of adsorption kinetics with the Avrami model, the fractal-like model, and the general-order kinetics model, as well as adsorption thermodynamics. This study fills the knowledge gap by presenting the preparation and characterization of activated carbon (L-AC) prepared from Mae Moh low-rank lignite by KOH chemical activation assisted with microwave heating. Specifically, the main goals are as follows: (i) to determine the optimal conditions for the synthesis of L-AC using various KOH impregnation ratios, microwave powers, and activation times; (ii) to study the physicochemical characteristics of L-AC using SEM, Raman, FTIR, and N_2_ adsorption–desorption measurements; (iii) to investigate the breakthrough performance of L-AC in the fixed-bed column at 298, 328, and 353 K; and (iv) to understand the kinetic adsorption mechanisms and intra-particle diffusion behavior, along with the adsorption thermodynamic parameters of CO_2_ uptake.

## 2. Results and Discussions

### 2.1. Morphological Characterization (SEM)

**Figure 1:**
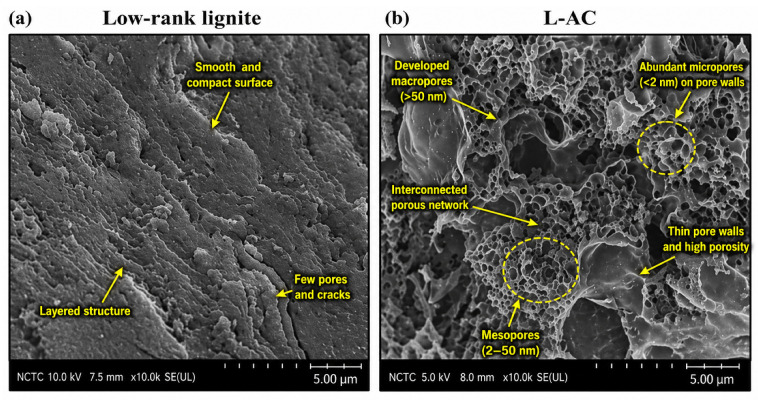
SEM image of (**a**) low-rank lignite and (**b**) L-AC at 10,000× Magnification.

The surface morphology of the raw low-rank lignite and L-AC was examined by SEM at 10,000× magnification, as shown in Figure 1. The inactivated low-rank lignite (Figure 1a) exhibits a smooth, tight, and largely non-porous surface with a distinct layering effect with very rare superficial cracks, thus indicating the high density and immature structural organization of the carbonaceous matrix of lignitic coal. It suggests the absence of surface area and porosity in the material and, hence, the unprocessed material is inherently incapable of acting as an adsorbent unless subjected to any form of treatment. The activated low-rank lignite (L-AC) (Figure 1b) exhibits a drastically altered hierarchy of porosity system comprising highly developed macroporosity (>50 nm), mesoporosity (2–50 nm), and an exceptionally large amount of microporosity (<2 nm) located on the pore walls and interconnectively arranged in a three-dimensional porous network. The macroporosity, created through gasification and volatilization during the pyrolysis and activation processes, serves as the transport route for the efficient delivery of the adsorbates into the interior regions of the particles. The notably thin pore walls and high overall porosity of L-AC maximize the fraction of material actively participating in adsorption while minimizing diffusional resistance. This well-developed hierarchical pore structure, rarely achieved without careful optimization of precursor and activation conditions, provides compelling morphological evidence for the superior adsorption performance of L-AC and underscores the effectiveness of the activation process in converting a low-cost lignite precursor into a high-performance carbonaceous adsorbent.

### 2.2. Structural Order and Graphitization (Raman Spectroscopy)

**Figure 2:**
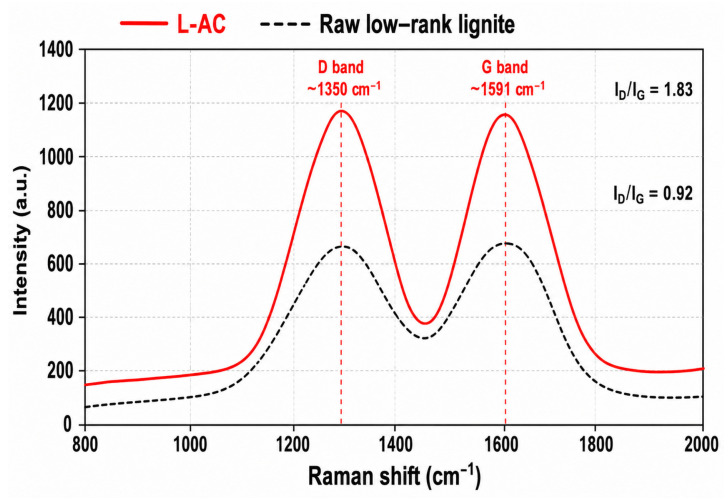
Raman spectra of raw low-rank lignite and L-AC (laser: 532 nm).

The structural order and degree of graphitization of raw low-rank lignite and L-AC were investigated by Raman spectroscopy, and the resulting spectra are presented in Figure 2. Both materials exhibited two characteristic first-order carbon bands in the region of 1000–1800 cm^−1^: the D band at approximately 1348 cm^−1^, associated with disordered sp^3^–hybridized carbon atoms and structural defects at the graphene layer edges (A_1_g symmetry mode), and the G band at approximately 1581 cm^−1^, attributed to the in-plane stretching vibration of sp^2^-hybridized carbon in graphitic crystallite domains (E_2_g mode) [23]. The intensity ratio I_D_/I_G_ serves as a quantitative index of structural disorder in carbonaceous materials, where higher values indicate greater defect density and reduced graphitic order [27]. The unprocessed low-rank lignite sample showed a relatively high I_D_/I_G_ value of 0.92, which suggests that it had a fairly disordered but reasonably well-organized carbon backbone characteristic of lignitic coal. Such a structure is characterized by partially formed aromatic ring structures in combination with aliphatic side chains, along with the presence of oxygen functional groups, as confirmed independently by FTIR spectroscopy in Section 2.3 [23]. The wide and slightly asymmetrical D and G bands in the spectrum of unprocessed lignite are indicative of its heterogeneity and immaturity. Following KOH chemical activation under microwave-assisted heating, the I_D_/I_G_ ratio of L-AC increased to 1.83, indicating a significantly higher density of structural defects, lattice discontinuities, and edge carbon atoms generated during the thermochemical activation process [21,28]. This increase in structural disorder is consistent with the well-established mechanism of KOH activation, in which chemical etching by potassium intercalation and gasification reactions preferentially disrupts ordered graphitic crystallite domains and creates abundant defect-rich micropore edge sites throughout the carbon matrix [21]. The elevated defect density in L-AC is directly correlated with the exceptional BET surface area of 1349 m^2^ g^−1^ and the dominant micropore population confirmed by the N_2_ adsorption–desorption analysis (Section 2.4), since defect sites and edge carbon atoms constitute the primary structural loci for pore nucleation and growth during KOH activation [28]. Furthermore, the higher I_D_/I_G_ ratio of L-AC provides additional evidence for enhanced CO_2_ adsorption affinity, as defect-rich edge carbon sites are known to strengthen the quadrupole–π interaction between CO_2_ molecules and the graphitic carbon surface, complementing the physisorptive mechanism proposed in Section 2.8 and Section 2.9 [21,28]. Taken together, the Raman spectroscopic evidence confirms that microwave-assisted KOH activation has successfully restructured the carbonaceous framework of low-rank lignite, generating a defect-enriched, high-surface-area activated carbon with physicochemical properties well-suited for efficient CO_2_ capture [21].

### 2.3. Surface Functional Groups (FTIR)

**Figure 3:**
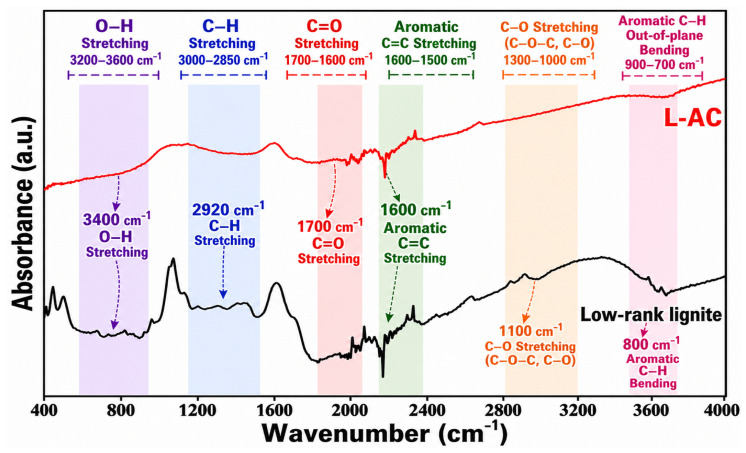
FT–IR spectra of low-rank lignite and L-AC.

The surface functional group compositions of the raw low-rank lignite and L-AC were characterized by Fourier-transform infrared (FTIR) spectroscopy, and the resulting spectra are presented in Figure 3. Based on the FTIR spectrum of the unprocessed low-rank lignite, it can be seen that there are many kinds of oxygen-containing and aliphatic functional groups. This indicates that the lignite material used as the precursor for activated carbon has a complex chemical composition and a relatively incomplete carbonization state. There is a strong absorption peak in the region of 3800–3600 cm^−1^. This is due to the existence of free O–H stretching vibrations, which show that there are surface-bound hydroxyl groups and structurally bound hydroxyl groups. It has been proven that, when the coal rank is low and the oxygen content is relatively high, such groups exist. In the region of 3000–2800 cm^−1^, there are aliphatic C–H stretching vibrations of methylene (–CH_2_–) and methyl (–CH_3_–) groups. This proves that there are aliphatic groups in the carbon lattice. There is an obvious absorption peak at 1596 cm^−1^, which is caused by aromatic ring C=C stretching. This also reflects the partial development of the aromatic structure in the lignite precursor. There are also absorption peaks at 1378 cm^−1^ and 1097 cm^−1^. The former is caused by the vibration of –CH_3_, and the latter is caused by aliphatic ether C–O–C stretching vibrations. This also proves that there are many oxygen-containing aliphatic groups in the precursor. The peak at 912 cm^−1^ corresponds to the bending vibration of O–H in carboxylic acid (–COOH) [29]. This is consistent with the high content of carboxyl groups in low-rank lignite coals. After the activation process, the FTIR spectrum of L-AC shows a significant decrease in functional group types, indicating that there has been considerable structural change and degradation of functional groups during the activation process. For example, the absorption peaks in the region of 3000–2800 cm^−1^ and the higher absorption peaks of hydroxyl groups have significantly weakened. This indicates that, during the activation process, aliphatic side chains and hydroxyl groups are removed through thermal decomposition reactions, such as pyrolysis and gasification. After the activation process, the two strongest absorption peaks in the FTIR spectrum of L-AC are located at 1046 and 1087 cm^−1^. These two absorption peaks are caused by the C–O and C=O stretching vibrations of carboxyl groups and ether groups. The existence of these oxygen-containing surface functional groups on the activated carbon is meaningful because these functional groups make the activated carbon surface more hydrophilic [2,22,30].

### 2.4. Textural Properties (BET, Pore Size Distribution, Surface Area, Pore Volume, Ultimate Analysis)

**Figure 4:**
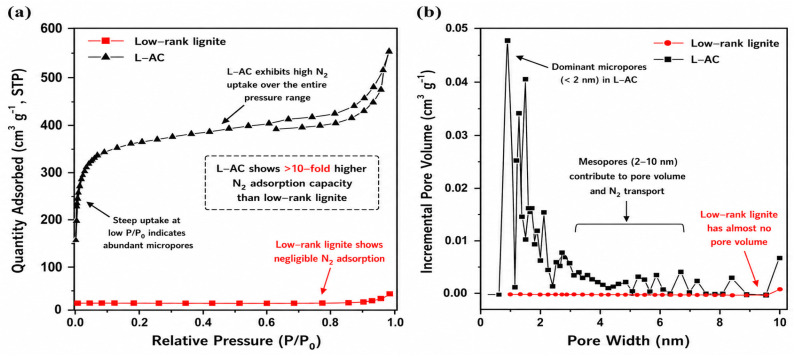
Low-rank lignite and L-AC characteristics: (**a**) N_2_ adsorption and desorption isotherm and (**b**) pore size distribution.

Figure 4 provides the quantitative characterization of the pore structures of the raw lignite precursor and the L-AC product via N_2_ adsorption–desorption isotherms and PSD. Figure 4a shows the essentially featureless N_2_ adsorption isotherm for raw lignite; in particular, there is essentially zero adsorption of N_2_ at any relative pressure ranging from 0 to 1. Such behavior indicates a nearly absolute absence of pores and porosity in general, meaning that the raw lignite sample has no accessible internal surface area and thus does not possess adsorption capability in either the gaseous or aqueous phase. In addition, the near-zero slope at low relative pressures indicates that there are hardly any micropores within the material, whereas the lack of hysteresis on the isotherm graph implies an absent mesopore system. Consistently, the compact layered surface microstructure visible in the SEM image (see Figure 1a) supports the conclusion that raw low-rank lignite, in its as-is state, cannot serve as a suitable adsorbent material.

By comparison, the N_2_ adsorption isotherm shown in Figure 4a indicates a quite different pattern of N_2_ uptake by the activated material. A marked steep initial increase is observed at relative pressures less than 0.1, meaning the presence of rich micropores in which strong adsorbent–adsorbate interactions cause fast pore-filling at lower equilibrium pressure values. According to the IUPAC classification, such behavior qualifies this isotherm as a type I curve, which is a clear sign of high microporousness [31]. After that, the isotherm displays a gradual increase in adsorption followed by another uptake jump at larger relative pressures, indicating that there are both micropores and mesopores in the material, with the latter starting to contribute to N_2_ adsorption at intermediate pressures. As clearly seen in Figure 4a, the total amount of N_2_ absorbed by L-AC is an order of magnitude larger than that absorbed by raw lignite, proving that the activation process has led to a drastic increase in the material’s total adsorption capacity due to the generation of internal pore spaces. Further quantitative insights about the internal porosity can be gained by analyzing the PSD results (Figure 4b). For raw lignite, the PSD curve appears essentially flat in the investigated pore width range of 1–10 nm; at the same time, the incremental pore volume of the material approaches zero value, implying the complete absence of both micropores and mesopores in the raw material. This observation agrees fully with the N_2_ adsorption data discussed above and, in conjunction with the microscopic SEM evidence of the non-porous character of raw lignite, provides solid proof of its non-porousness.

Unlike raw lignite, the PSD of L-AC shows a sharp peak for micropores with pore widths less than 2 nm and a pronounced micropore maximum corresponding to pore diameters of about 1–2 nm. Such peak in the PSD is clear evidence of an effective generation of micropores by the activation treatment, resulting in a high BET surface area and a large total amount of N_2_ absorbed. In particular, it should be noted that the sharpness of the micropore maximum implies relative uniformity of the micropore distribution, which can be advantageous when dealing with selective adsorption applications. Besides the micropores dominating the PSD spectrum, some contributions from mesopores with diameters of 2–10 nm are also observed. In other words, there are two pore types within the L-AC structure micropores, which are responsible for the adsorption capacity, and mesopores, which facilitate mass transport. The presence of the mesopore fraction is essential for overcoming the intrinsic kinetic limitations inherent to microporous adsorbents and achieving a high mass transfer rate during practical operation.

Overall, the N_2_ adsorption–desorption isotherms and PSD shown in Figure 4 indicate the successful development of a hierarchical micropore–mesopore structure in L-AC through activation treatment of raw low-rank lignite. The coexistence of highly developed micropore adsorption sites along with a mesopore network allowing efficient intra-particle mass transfer constitutes an ideal adsorbent texture. The order-of-magnitude enhancement in the N_2_ uptake capacity and the pronounced micropore maximum in the PSD clearly suggest that the activation procedure has unlocked the inherent adsorption capacity of the lignite precursor, making it a powerful adsorbent capable of handling challenging environmental remediation tasks.

**Figure 5:**
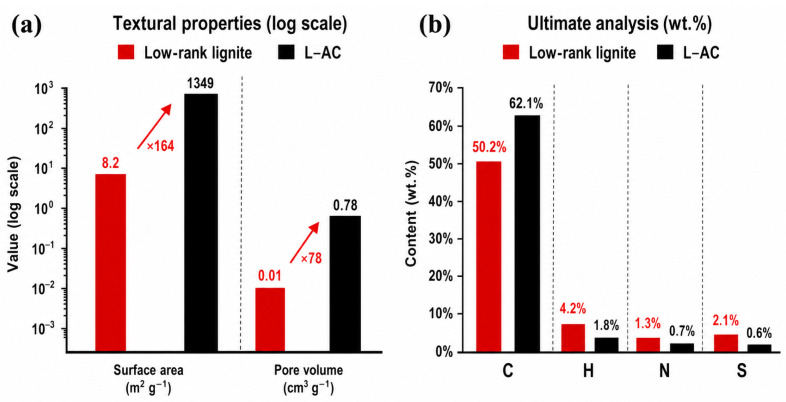
Surface area, pore volume, average pore size, and ultimate analysis of low-rank lignite and L-AC: (**a**) textural properties (log scale) and (**b**) ultimate analysis (wt.%).

The textural and elemental characteristics of the raw low-rank lignite and L-AC are provided in Figure 5 and Table 1 below, respectively, the detailed analysis of which provides compelling physicochemical evidence of the transformation caused by the activation process. The BET specific surface area value of the raw low-rank lignite was measured to be equal to 8.2 m^2^ g^−1^, which is consistent with its highly compact and non-porous microstructure visualized by SEM and the lack of any N_2_ adsorption on this carbon precursor in the isotherm experiment. The BET specific surface area value of the L-AC sample increased dramatically after activation, reaching 1349 m^2^ g^−1^, i.e., being 165 times higher than that of the starting material. Such drastic change in the surface area is the best evidence of successful transformation, which resulted in the formation of numerous micropore and mesopore structures in the course of the thermochemical modification of the carbon matrix. Noteworthy is that the increase in the total pore volume, going from 0.01 cm^3^ g^−1^ in the raw low-rank lignite to 0.78 cm^3^ g^−1^ in the L-AC, was almost as remarkable, amounting to 78 times larger than the initial value. The simultaneous and proportional increase in the surface area and pore volume values, as well as the high specific values themselves, suggest that L-AC is a promising carbonaceous adsorbent derived from the carbon precursor, which cannot be used as such due to the absence of porosity. The average pore size values of the examined carbon samples offer further evidence of the nature of the structural changes, occurring in the material during activation. The value of this indicator for the raw low-rank lignite was found to be 89.8 nm, which corresponds to the macroporous category (macropores > 50 nm) of the IUPAC pore size classification system. Thus, the rather high value of the average pore diameter together with the extremely low total pore volume indicates that the small number of pores in the raw lignite are predominantly macropores of a relatively large size, which hardly provide any surface area for adsorption. On the contrary, the L-AC sample has a much smaller value of average pore diameter, i.e., 5.61 nm, being 16 times lower than that of the precursor, and thus belonging to the mesoporous (2–50 nm) category. This transition from macroporous to mesoporous pore size distribution, combined with abundant creation of micropores confirmed by the PSD graph shown in Figure 5, shows that activation has led to the formation of a hierarchically structured porous carbon matrix, which is optimal for high-capacity adsorption.

The ultimate analysis results of the two carbon samples reported in Table 1 offer additional evidence of the transformation of the carbonaceous matrix. As seen from the table, there was a substantial rise in the carbon content from 50.2 wt% in the raw low-rank lignite to 62.1 wt% in L-AC, implying that the pyrolysis-activation process resulted in the elimination of other heteroatoms in favor of carbon atoms. Namely, there was an intensive volatilization of oxygen, hydrogen, nitrogen, and sulfur species, forming the vast number of functional groups on the carbon matrix during heating at elevated temperatures. Such an increase in carbon content can be explained by the thermal carbonization process, leading to the decomposition of organic side chains and the elimination of oxygen-containing moieties, resulting in the formation of aromatic fragments in the carbon matrix. As a consequence, the hydrogen content was reduced drastically, going from 4.2 wt% in raw low-rank lignite to 1.8 wt% in the modified material. Such a decrease in the aliphatic hydrogen content is confirmed by the attenuation of C–H stretching vibrations in the FTIR spectrum of L-AC (see Figure 3). The nitrogen content also slightly decreased from 1.94 to 1.62 wt%. At the same time, the sulfur content decreased significantly, decreasing from 2.52 to 0.60 wt%. The latter result can be explained by the fact that sulfurous species in carbonaceous materials can negatively affect the performance of the material and interfere with its adsorption selectivity. Thus, the significant decrease in sulfur content, which decreased by almost threefold during activation, indicates the effective removal of these impurities at high temperatures. The general decrease in H, N, and S contents, and the increase in the content of carbon are indicative of an elevated degree of carbonization and aromatization of the carbon material.

Considered together, the textural and elemental data presented in Figure 5 and Table 1 provide mutually reinforcing evidence that the activation of low-rank lignite has produced a material L-AC with a dramatically enhanced specific surface area, a well-developed hierarchical pore structure, and a chemically enriched carbonaceous framework. The 165-fold increase in the BET surface area, the 78-fold increase in the total pore volume, the 16-fold reduction in the average pore size, and the concurrent increase in the carbon content collectively confirm the successful conversion of a structurally and chemically immature lignite precursor into a high-performance activated carbon with physicochemical properties well-suited for demanding adsorption-based environmental applications.

To further evaluate the structural characteristics of the developed L-AC, its textural properties were compared with representative coal- and biomass-derived activated carbons reported in previous studies (Table 2). The BET surface area of L-AC (1349 m^2^ g^−1^) is comparable to or higher than many conventional activated carbons prepared from lignite, bituminous coal, coconut shell, and agricultural biomass using thermal or chemical activation methods. In addition, the hierarchical microporous–mesoporous structure and relatively large pore volume are expected to facilitate rapid CO_2_ diffusion and to improve adsorption efficiency under dynamic fixed-bed conditions. These comparisons demonstrate that microwave-assisted KOH activation effectively converts low-rank lignite into a highly porous carbon adsorbent with competitive structural properties.

It should be noted that the pore structure of L-AC was characterized using N_2_ adsorption–desorption at 77 K, which is the standard method for determining the textural properties of porous carbon materials. Although this technique provides reliable information on the specific surface area, pore volume, and pore-size distribution, complementary CO_2_ adsorption measurements would provide greater sensitivity for characterizing ultra-micropores (<0.7 nm), which are particularly important for CO_2_ adsorption. Therefore, future work will incorporate CO_2_ adsorption porosimetry to further elucidate the contribution of ultra-micropores to the adsorption performance of L-AC.

### 2.5. CO_2_ Breakthrough Adsorption Performance

#### 2.5.1. Breakthrough Curves at Varying Temperature

**Figure 6:**
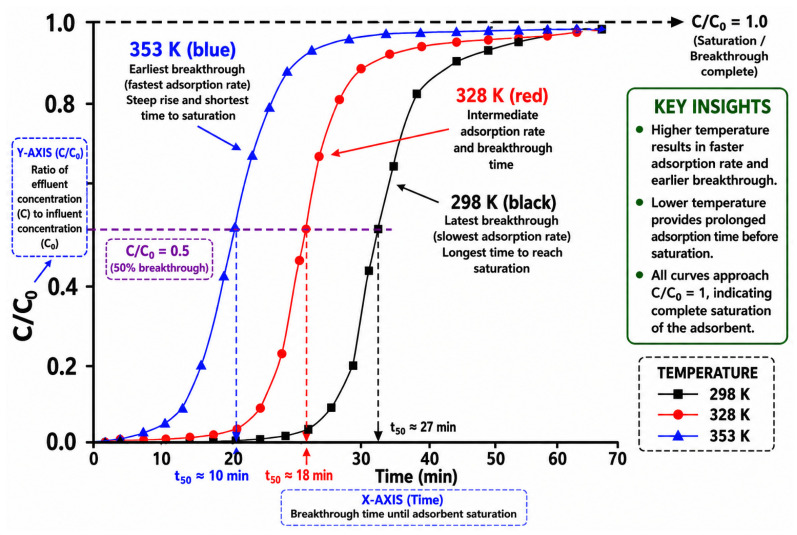
The CO_2_ adsorption breakthrough curve of L-AC at various temperatures.

The effect of operating temperature on the dynamic CO_2_ capture performance of L-AC was systematically investigated under atmospheric pressure (1 atm) using a fixed gas flow rate of 50 mL min^−1^ with an initial CO_2_ concentration of 15 vol% balanced in N_2_. Breakthrough experiments were conducted at three temperatures 298, 328, and 353 K and the resulting breakthrough curves are presented in Figure 6.

There is also an evident inverse correlation between the operating temperature and the CO_2_ adsorption retention time on L-AC. In particular, at 298 K, the adsorption curve rises the slowest, with the half-saturation time (*t*_50_) equal to approximately 27 min. It indicates that the lowest temperature causes the highest CO_2_ retention period, and thus the slowest adsorption rate, with the adsorbent becoming saturated with CO_2_ the latest at this temperature out of the examined cases. When the temperature was raised to 328 K, *t*_50_ drops down to 18 min; at 353 K *t*_50_ equals 10 min with the sigmoid curve leading towards saturation (*C*/*C*_0_ = 1.0) at the fastest pace. Moreover, the breakthrough time, defined as the moment when the concentration of CO_2_ starts rising significantly after being equal to zero in the initial state, is reduced from 15 min (298 K) to 9 min (353 K), along with the increase in the operating temperature. All tested breakthrough curves converged into the saturation point (*C*/*C*_0_ = 1.0) in the end. The gradual decrease in both t_50_ and breakthrough times observed with temperature elevation is a strong indication that CO_2_ adsorption onto L-AC is an exothermic process that occurs mainly via physisorption [15,26]. Namely, the adsorption is based on the weak, reversible van der Waals forces between the carbon surface and CO_2_ molecules instead of strong covalent or ionic bonds. With rising temperature, the adsorbed molecules gain more kinetic energy that helps them to break off the weak surface bonds, thereby making desorption prevail over adsorption [2]. Such behavior is consistent with the micropore structure of L-AC, proven via BET and PSD analyses, since the physisorption taking place within narrow micropores becomes highly dependent on the temperature due to shallow potential energy wells formed by van der Waals forces. Therefore, lower temperatures should be preferred when using L-AC for capturing CO_2_ in practice.

#### 2.5.2. Thomas and Yoon–Nelson Breakthrough Curve Modeling

**Figure 7:**
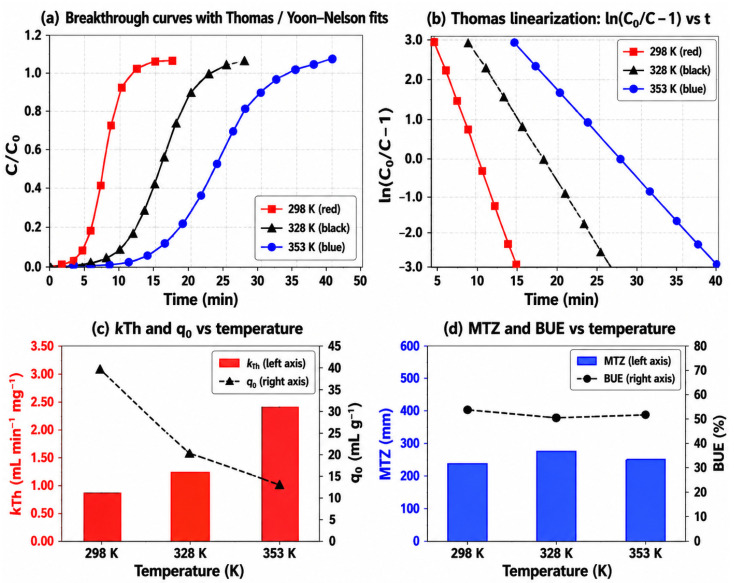
CO_2_ adsorption performance and kinetics on L-AC at differences temperatures; Effect of temperature on CO_2_ adsorption breakthrough behavior, kinetic parameters, and bed performance metrics.

To quantitatively characterize the dynamic CO_2_ adsorption behavior of L-AC in the fixed bed, the experimental breakthrough curves at 298, 328, and 353 K were analyzed using the Thomas model and the Yoon–Nelson model. The fitted parameters are summarized in Table 3 and Figure 7.

The Thomas model, one of the most widely applied expressions for fixed-bed adsorption column design, assumes plug flow, Langmuirian adsorption–desorption equilibrium, and negligible axial dispersion. The model is expressed as follows:

(1) $$\frac{C}{C_{0}}=\frac{1}{1\;+\;\exp(\frac{k_{Th}q_{0}m}{Q}-k_{Th}C_{0}t)}$$

where *k_Th_* (mL mg^−1^ min^−1^) is the Thomas rate constant, *q*_0_ (mg g^−1^) is the maximum adsorption capacity predicted by the model, *m* is the adsorbent mass (g), *Q* is the volumetric flow rate (mL min^−1^), *C*_0_ is the inlet CO_2_ concentration (mg mL^−1^), and *t* is time (min). The parameters were estimated by linearizing the model as ln(*C*_0_/*C* − 1) = *k_Th_q*_0_*m*/*Q* − *k_Th_C*_0_*t* and applying linear regression over the 5–95% breakthrough region (*R^2^* = 0.9996–0.9998).

Thomas’s rate constant *k_Th_* showed a monotonically increasing behavior with an increase in temperature, starting from 0.9075 mL mg^−1^ min^−1^ at 298 K and reaching 2.5799 mL mg^−1^ min^−1^ at 353 K. Such behavior can be explained by an increased molecular mobility and faster attainment of equilibrium at high temperatures. On the other hand, the value of Thomas’s adsorption capacity *q*_0_ dropped from 135.00 mg g^−1^ at 298 K to 50.00 mg g^−1^ at 353 K, which is consistent with the exothermic nature of physisorption found in Section 2.5 and Section 2.8. The values of the Thomas adsorption capacity q_0_ appear larger than those of the experimentally obtained capacity *q_e_* (47.34 − 21.34 mg g^−1^) for all temperatures considered. Such a difference is normal, since q_0_ is the ideal theoretical value and *q_e_* is a practically limited one.

The Yoon–Nelson model, which requires no assumptions about adsorbate properties or bed characteristics, describes the breakthrough as follows:

(2) $$\frac{C}{C_{0}}=\frac{1}{1\;+\;\exp(k_{YN}(\tau_{50}-t))}$$

where *k_YN_* (min^−1^) is the Yoon–Nelson rate constant and *τ*_50_ (min) is the time required for 50% adsorbent breakthrough. The *k_YN_* values increased from 0.2450 min^−1^ at 298 K to 0.5880 min^−1^ at 353 K, confirming that the rate of bed saturation accelerates with increasing temperature. The *τ*_50_ values (27, 18, and 10 min at 298, 328, and 353 K, respectively) are in excellent agreement with the experimentally observed *t*_50_ values reported in Section 2.6.1, validating the accuracy of the model fit.

The mass transfer zone (MTZ) length, calculated as MTZ = *L* × (1 − *t_b_*/*t_s_*), where *L* is the bed length, *t*b the breakthrough time, and *t*s the saturation time, was 246.2 mm at 298 K, and increased to 266.7 mm at 328 and 353 K. The broad MTZ relative to the total bed length (400 mm) indicates that a substantial fraction of the bed is engaged in active mass transfer at any given time, confirming that external film diffusion and mesopore transport identified as rate-limiting steps by the Weber–Morris analysis (Section 2.6.2) are the dominant resistances governing the axial concentration profile. The bed utilization efficiency (BUE = *q_e,exp_*/*q*_0_ × 100%) increased from 34.9% at 298 K to 43.2% at 353 K, reflecting the narrower MTZ relative to the overall bed capacity at higher temperatures, where mass transfer kinetics are faster even as equilibrium capacity declines. The relatively low BUE at 298 K, despite the highest *q_e_*, indicates that the slow mass transfer at lower temperatures prevents full utilization of the available adsorption capacity before breakthrough occurs, suggesting that bed length optimization or reduced superficial velocity could improve BUE under ambient temperature operation.

### 2.6. Adsorption Kinetics

#### 2.6.1. Kinetic Model Fitting

**Figure 8:**
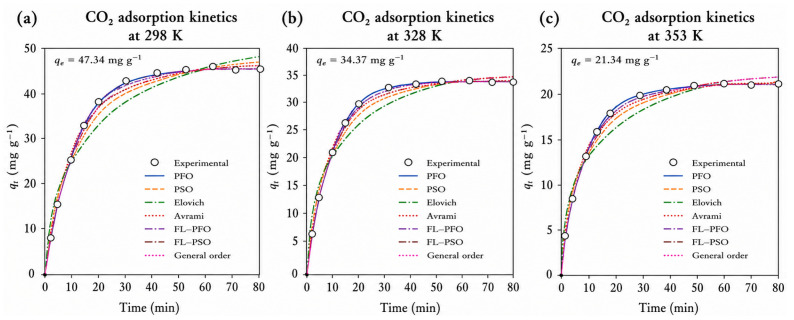
Kinetic adsorption for CO_2_ adsorbed onto the L-AC adsorbent: (**a**) 298 K, (**b**) 328 K, and (**c**) 353 K.

Seven kinetic models were fitted to the experimental CO_2_ uptake data at 298, 328, and 353 K; the resulting parameters are summarized in Table 4 and the corresponding curves are shown in Figure 8a–c. The PFO model gave good fit with *R^2^* = 0.9987–0.9995 and *SSE* = 0.77–1.62, although slight deviation from the experimental points was still observed at longer adsorption times. The PSO model showed lower fitting accuracy than PFO, with *R^2^* = 0.9900–0.9948 and *SSE* = 3.15–31.02, indicating that the pseudo-second-order chemisorption was not the dominant rate-limiting mechanism. The Elovich model provided the weakest fit among the models, with *R^2^* = 0.9634–0.9690 and *SSE* = 19.37–113.71, confirming that a simple heterogeneous chemisorption description was not suitable for CO_2_ adsorption onto L-AC. By contrast, the Avrami model showed excellent agreement with the experimental data, with *R^2^* = 0.9995–0.9998 and *SSE* = 0.19–1.62. The FL-PFO model also provided an excellent fitting performance, with *R^2^* = 0.9995–0.9998 and *SSE* = 0.19–1.62, confirming the contribution of time-dependent heterogeneous adsorption kinetics. The FL-PSO model gave acceptable but slightly weaker fitting, with *R^2^* = 0.9960–0.9984 and *SSE* = 0.99–12.39. Finally, the general-order model also showed excellent statistical performance, with *R^2^* = 0.9994–0.9997 and *SSE* = 0.21–1.72. The predicted equilibrium capacities were close to the revised values of 47.34, 34.37, and 21.34 mg g^−1^ at 298, 328, and 353 K, respectively. Overall, the Avrami, FL-PFO, and general-order models best described the CO_2_ adsorption kinetics of L-AC.

The CO_2_ adsorption performance of L-AC was further compared with representative activated carbons reported in the literature under similar post-combustion conditions (as shown in Table 2). Although the adsorption capacity depends on experimental conditions, such as CO_2_ concentration, temperature, and pressure, the developed L-AC exhibited a competitive adsorption performance while being synthesized from abundant low-rank lignite using a rapid microwave-assisted activation process. Compared with many previously reported coal- and biomass-derived activated carbons, L-AC combines a high specific surface area, hierarchical pore structure, defect-rich carbon framework, and excellent cyclic stability, demonstrating its potential as a low-cost and scalable adsorbent for post-combustion CO_2_ capture.

#### 2.6.2. Intra-Particle Diffusion

The Weber–Morris intra-particle diffusion presents the corresponding parameters summarized in Table 5. Instead, three distinct diffusion regions were observed, indicating that CO_2_ adsorption onto L-AC proceeded through a multi-stage transport mechanism involving external film diffusion, intra-particle diffusion, and equilibrium adsorption. The first stage *t*^0^.^5^ = 1.0–2.8 min^0.5^ exhibited relatively high $k_{id1}$ values of 7.52, 5.42, and 3.68 mg g^−1^ min^0.5^ at 298, 328, and 353 K, respectively, corresponding to the rapid transfer of CO_2_ molecules from the bulk gas phase to the external surface of L-AC. The second stage (*t*^0.5^ = 3.0–7.0 min^0.5^) represented diffusion within the internal mesoporous/microporous structure and acted as the primary rate-controlling step, with $k_{id2}$ values of 7.84, 6.08, and 4.41 mg g^−1^ min^−0.5^, respectively. In the third stage (*t*^0.5^ = 7.0–9.0 min^0.5^), the very low slope values (*k_id_*_3_ = 0.52–0.19 mg g^−1^ min^−0.5^) and high positive intercepts (*C*_3_ = 20.77–46.30 mg g^−1^) indicated gradual saturation of adsorption sites and equilibrium establishment. Furthermore, both the diffusion rate constants and equilibrium adsorption capacities decreased with the increasing temperature, where $q_{e}$ decreased from 47.34 mg g^−1^ at 298 K to 21.34 mg g^−1^ at 353 K, confirming the exothermic physisorption nature of CO_2_ adsorption onto L-AC. The persistence of non-zero intercepts in all regions further demonstrated that external film diffusion also contributed to the overall adsorption mechanism.

The three-stage Weber–Morris behavior of L-AC is consistent with recent reports on hierarchically porous carbonaceous CO_2_ adsorbents. The Stage I rate constants (*k_id1_* = 7.52–3.68 mg g^−1^ min^−0.5^, 298–353 K) are comparable to those of KOH-activated olive-waste carbon (7.2–11.4 mg g^−1^ min^−0.5^) and palm shell AC (6.8–10.1 mg g^−1^ min^−0.5^), confirming that boundary-layer transport in L-AC is within the range expected for microporous carbons of similar particle dimensions. The Stage II constants (*k_id2_* = 7.84–4.41 mg g^−1^ min^−0.5^) align with those of coal-based GAC (0.4–4.9 mg g^−1^ min^−0.5^), and Stage II is identified as the primary rate-limiting step in both systems. The near-zero *k_id_*_3_ values (0.52–0.19 mg g^−1^ min^−0.5^) are consistent with the micropore saturation constants reported for activated hydrochar (≈0.08–0.30 mg g^−1^ min^−0.5^). The monotonic decline of all *k_id_* values with temperature attributable to the dominant reduction in equilibrium driving force rather than retarded molecular mobility has been independently reported for GAC in syngas CO_2_ removal and for CO_2_ adsorption on Malaysian coal. A key distinction of this study is the simultaneous application of seven kinetic models alongside the IPD analysis; whereas most of the literature studies have relied on the PFO, PSO, and Avrami models alone, the general-order and FL-PFO models employed here delivered a statistically superior fit (*R^2^* ≥ 0.9989; *SSE* ≤ 3.21), providing a more rigorous characterization of the surface heterogeneity and time-dependent kinetics of L-AC. As summarized in Table 6, the IPD parameters of L-AC are quantitatively competitive with recently published carbonaceous adsorbents, while the high *k_id1_* and low *k_id_*_3_ jointly indicate rapid initial uptake with complete micropore utilization, a combination favorable for temperature-swing adsorption (TSA) applications.

### 2.7. Adsorption Mechanism

#### 2.7.1. Three-Stage Transport Mechanism (IPD)

**Figure 9:**
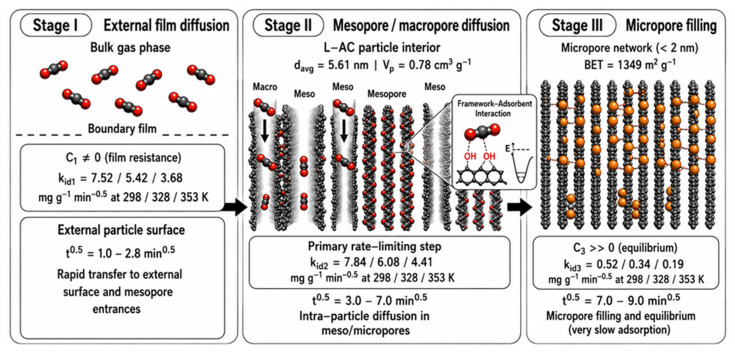
CO_2_ adsorption mechanism on L-AC (Image generated by ChatGPT Plus and Claude AI Pro, 2026).

The mechanism governing CO_2_ adsorption onto L-AC is proposed on the basis of the integrated evidence from the morphological characterization (SEM), textural analysis (BET/BJH), surface chemistry (FTIR), kinetic modeling, and intra-particle diffusion analysis, as schematically illustrated in Figure 9. The adsorption process proceeds through three sequential and mechanistically distinct transport stages, collectively described by the Weber–Morris intra-particle diffusion (IPD) model, before culminating in the establishment of thermodynamic equilibrium within the micropore network.

In Stage I (*t* < 24 min), CO_2_ molecules in the bulk gas phase present at an initial concentration of 15 vol% in N_2_ migrate across the stagnant boundary layer surrounding each L-AC particle under the influence of a steep concentration gradient. The diffusion in the film, characterized by *k_id1_* values of 7.52, 5.42, and 3.68 mg g^−1^ min^−0.5^ at 298, 328, and 353 K respectively, happens quite quickly due to easy access to the external surface of L-AC, coupled with the maximum concentration gradient within the film at the beginning of each adsorption run. During the second phase, when 24 min < *t* < 42 min, the CO_2_ gas that passes through the external film moves further to diffuse into the interior structure of L-AC with hierarchical porosity, including macropores (transport channels) and mesopores (*d_avg_* = 5.61 nm; *V_p_* = 0.78 cm^3^ g^−1^). The intra-particle diffusion process demonstrates significantly lower rate constant values (*k_id2_* = 4.41–7.84 mg g^−1^ min^−0.5^) due to greater resistance in diffusing CO_2_ gases through the complex pore wall network, whereby available surface sites gradually fill up. Phase II is considered to be the main limiting step of the adsorption process due to the control it exerts on the supply of CO_2_ to the micropore surface.

During the third stage (*t* > 42 min), CO_2_ molecules reach the densely packed network of micropores (<2 nm), contributing most significantly to the exceptional BET surface area of L-AC (1349 m^2^ g^−1^). The interactions between the CO_2_ molecules and the carbon surfaces of the micropores are purely physical in nature, involving the process of van der Waals forces-mediated reversible physisorption with the contribution from the residual oxygen-containing functional groups as detected by the FTIR analysis through the presence of the C=O stretching vibration at 1046 cm^−1^ and the C–O stretching vibration at 1087 cm^−1^. These functional groups have been shown to increase the interaction between the quadrupole moment of the CO_2_ molecule and the carbon surfaces. The near-zero values of the rate constants (*k_id_*_3_ = 0.19–0.52 mg g^−1^ min^−0.5^) and positive intercepts (*C*_3_ = 20.77–46.30 mg g^−1^) confirm the exhaustion of the micropore adsorption sites and the achievement of thermodynamic equilibrium at *q_e_* = 47.34, 34.37, and 21.34 mg g^−1^ at 298, 328, and 353 K, respectively. The decreasing trend of adsorption capacities with increasing temperature demonstrates the exothermic nature of the physisorption process of CO_2_ on L-AC. Unlike chemisorption, where directional and specific covalent/ionic bonds dictate the interactions between the adsorbate and the adsorbent, under physisorption conditions, the adsorbate–adsorbent interactions are dominated by weak and non-directional van der Waals forces. As such, with an increase in temperature, the adsorbed molecules will have more kinetic energy to escape out of the shallow potential energy wells of the van der Waals forces and thus drive the adsorption–desorption equilibrium towards desorption. The dependence of adsorption capacities on temperature is therefore a clear indication of the predominantly microporous structure of L-AC. In microporous structures, the potential field overlaps between the opposing walls within the pores produces a strong adsorption potential, but not sufficiently strong enough to keep CO_2_ in its physical state under high temperatures.

**Figure 10:**
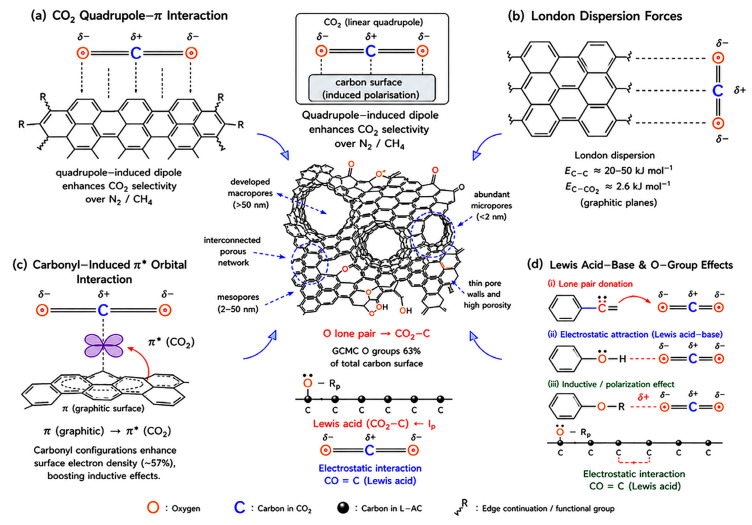
Proposed adsorption mechanism of CO_2_ onto KOH-activated lignite carbon (L-AC): molecular interactions at the graphitic surface, oxygen-containing functional groups, and micropore confinement corroborated by SEM, FTIR, and intra-particle diffusion analysis (Image generated by ChatGPT and Claude AI, 2026).

Hence, low temperatures (≤298 K) are recommended to enhance the adsorption capacities of L-AC in CO_2_ capture processes. Additionally, the reversibility of the adsorption interaction is confirmed by the retention of the adsorption capacity across six adsorption–desorption cycles, implying that neither the microporous structure nor the oxygen-containing surface functional group compositions of L-AC changes during thermal regeneration for TSA applications. The adsorption of CO_2_ onto L-AC is governed by reversible physisorption mediated through four synergistic molecular-level interactions, as illustrated in Figure 10. The dominant contribution arises from London dispersion forces between CO_2_ molecules and the graphitic π–electron planes of L-AC (E_C–C_ ≈ 20–50 kJ mol^−1^; E_C–CO2_ ≈ 2.6 kJ mol^−1^), driven by the high polarizability of CO_2_ (*α* = 2.65 Å^3^) and the extended aromatic carbon network developed upon KOH activation [45].

The proposed adsorption mechanism is based on integrated evidence from SEM, BET/BJH, FTIR, Raman spectroscopy, adsorption kinetics, intra-particle diffusion, thermodynamic analysis, and the relevant literature. Although these complementary results consistently support the contributions of defect-rich carbon structures, oxygen-containing functional groups, Lewis acid–base interactions, CO_2_ quadrupole–π interactions, and van der Waals forces, they do not provide direct molecular-level evidence of the adsorption process. Therefore, the proposed mechanism should be regarded as a plausible interpretation supported by the available experimental observations. Future studies employing advanced in situ spectroscopic techniques (e.g., DRIFTS and XPS) together with density functional theory (DFT) calculations will further elucidate the interactions between CO_2_ molecules and the active adsorption sites of L-AC.

A second, selectivity-determining interaction arises from the large linear quadrupole moment of CO_2_ (Θ = −4.3 × 10^−40^ C·m^2^), which generates a quadrupole-induced dipole interaction with the polarizable carbon surface. Because Θ(CO_2_) substantially exceeds that of N_2_ (Θ = −1.4 × 10^−40^ C·m^2^) and is essentially absent in CH_4_ (Θ ≈ 0), this interaction confers the inherent thermodynamic selectivity of L-AC for CO_2_ over competing flue gas components [49]. Specifically, the electrostatic contribution derives from the residual oxygen-containing surface functional groups that have been characterized by FTIR spectroscopy as C=O stretching at 1046 cm^−1^ and C–O–C stretching at 1087 cm^−1^. Since the carbon center in CO_2_ is electron-deficient (Lewis acid, *δ^+^*), a directed electrostatic effect takes place due to the acceptance of lone-pair electrons by the carbon center from surface carbonyl and ether oxygen atoms. The GCMC simulation data confirm that oxygen-containing surface groups are responsible for about 63% of total CO_2_ uptake by similar carbonaceous surfaces. Furthermore, the π* orbital character of the C–O ether group results in the enhancement of surface electron density by ~57% at adsorption sites, which contributes to the inductive effect [49].

Another important structural feature that increases all four molecular interactions is the hierarchical micropore–mesopore structure of L-AC (the BET surface area is equal to 1349 m^2^ g^−1^; the pore volume *V_p_* = 0.78 cm^3^ g^−1^; the average pore diameter *d_avg_* = 5.61 nm) depicted in the SEM micrograph. It is well known that in micropores smaller than approximately 2 nm the total effect of overlapped van der Waals potential fields between opposite walls increases the adsorption energy compared to the flat surface by a factor of 1.5–2. The macropore–mesopore–micropore transport system, including macropores (d > 50 nm) as main diffusion pathways, mesopores (2 < d < 50 nm) as second diffusion channels, and micropores (d < 2 nm) as adsorption sites, provides a mechanism behind not only high equilibrium capacity (*q_e_* = 47.34 mg g^−1^ at 298 K) but excellent desorbate reusability up to ten adsorption–desorption cycles. This phenomenon is explained by the physisorption of CO_2_ and its exothermic nature (Δ*E_a_* ≈ 23 kJ mol^−1^).

#### 2.7.2. Chemical Reaction Pathways of CO_2_ Adsorption

**Figure 11:**
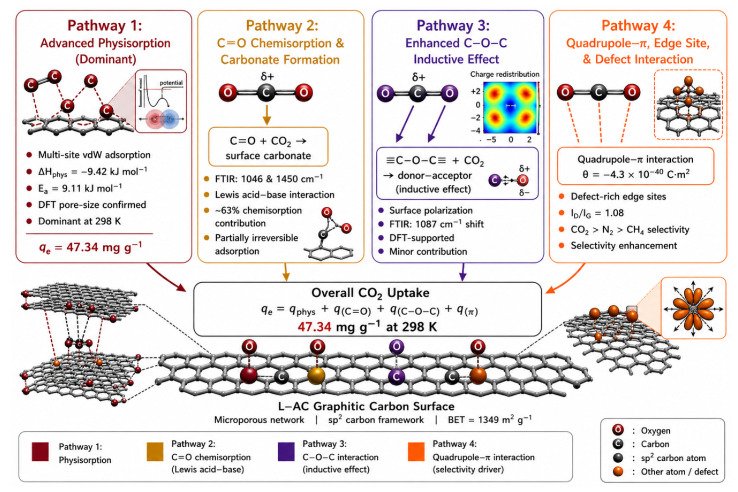
Chemical reaction pathways of CO_2_ adsorption on L-AC: (1) physisorption via van der Waals dispersion forces; (2) chemisorption via surface carbonyl groups (C=O, FTIR: 1046 cm^−1^) through Lewis acid–base interaction; (3) donor–acceptor complex formation via surface ether groups (C–O–C, FTIR: 1087 cm^−1^); and (4) quadrupole–π interaction at graphitic defect sites (Raman: I_D_/I_G_ = 1.83). The overall equilibrium capacity *q_e_* integrates all four contributions (Image generated by ChatGPT and Claude AI, 2026).

The CO_2_ adsorption process on L-AC proceeds through both physisorption and chemisorption pathways (Figure 11), governed by the surface chemistry identified by the FTIR (Section 3.3) and the micropore architecture confirmed by the BET analysis (Section 3.4). The dominant physisorption pathway involves reversible van der Waals interactions, while the minor chemisorption contribution arises from the residual oxygen-containing surface functional groups, specifically carbonyl (C=O) and ether (C–O–C) groups reacting chemically with adsorbed CO_2_ molecules. The full reaction sequence is described below.

Pathway 1—Physisorption (dominant pathway):

The primary adsorption mechanism is physical entrapment of CO_2_ within the micropore network through van der Waals dispersion forces, which can be expressed as follows:

(3) $${\mathrm{CO}}_{2}\left(g\right)\overset{\mathrm{van}\;\mathrm{der}\;\mathrm{Waals}}{\to }{\mathrm{CO}}_{2}\left(\mathrm{ads}\right)\;\;\Delta H\;=\;-9.42\;{\mathrm{kJ}\;\mathrm{mol}}^{-1}\;(\mathrm{exothermic},\;\mathrm{reversible})$$

This is confirmed by the thermodynamic data (Δ*H°* < 40 kJ mol^−1^) and near-complete regeneration over 10 cycles.

Pathway 2—Chemisorption via surface carbonyl groups (C=O):

The electron-deficient carbon center of CO_2_ (Lewis acid, *δ^+^*) interacts with the lone-pair electron density of surface carbonyl oxygen atoms (Lewis base), forming a surface carbonate–like intermediate, which can be expressed as follows:

(4) $$\equiv C=O+{\mathrm{CO}}_{2}\to \;\equiv C=O\cdot \cdot \cdot {\mathrm{CO}}_{2}\to \equiv C–O–{\mathrm{COO}}^{\delta -}$$

Or can be written as a formal surface carbonate formation as follows:

(5) $$C_{\mathrm{surface}}\;=\;\;O+{\mathrm{CO}}_{2}\to C_{\mathrm{surface}}–O–C(=O)–O^{\delta -}$$

This interaction is electrostatic and partially reversible, consistent with the low activation energy (*E_a_* = 9.11 kJ mol^−1^) observed in the Arrhenius analysis (Section 2.8).

Pathway 3—Chemisorption via surface ether groups (C–O–C):

The ether oxygen acts as an electron donor toward the electrophilic carbon of CO_2_, forming a weak donor–acceptor complex, which can be expressed as follows:

(6) $$\equiv C–O–C\equiv \;+\;{\mathrm{CO}}_{2}\to \;\equiv C–O–C\equiv \cdot \cdot \cdot {\mathrm{CO}}_{2}$$

(7) $$\equiv {C–O}^{\delta -}\cdot \cdot \cdot C^{\delta +}=O\cdot \cdot \cdot O^{\delta -}$$

This inductive interaction, identified by the C–O stretching band at 1087 cm^−1^ in the FTIR spectrum, enhances local surface electron density at adsorption sites and strengthens the overall CO_2_-surface binding energy without forming a stable covalent bond.

Pathway 4—Quadrupole–π interaction at graphitic defect sites:

At defect-rich edge carbon sites confirmed by Raman spectroscopy (I_D_/I_G_ = 1.83, Section 3.2), the large linear quadrupole moment of CO_2_ (Θ = −4.3 × 10^−40^ C·m^2^) interacts with the π–electron cloud of sp^2^-hybridized graphitic domains, which can be expressed as follows:

(8) $${\mathrm{CO}}_{2}\left(g\right)+\pi_{\mathrm{graphene}}\to [{\mathrm{CO}}_{2}\cdot \cdot \cdot \pi {]}_{\mathrm{ads}}$$

$$\Theta_{{\mathrm{CO}}_{2}}\gg \Theta_{N_{2}}\;\Rightarrow \;{\mathrm{inherent}\;\mathrm{selectivity}\;\mathrm{of}\;\text{L-AC}\;\mathrm{for}\;\mathrm{CO}}_{2}{\;\mathrm{over}\;N}_{2}$$

This interaction is purely physical but directional, conferring thermodynamic selectivity over N_2_ (Θ = −1.4 × 10^−40^ C·m^2^) and CH_4_ (Θ ≈ 0).

Overall combined adsorption equilibrium expression, the net CO_2_ uptake on L-AC integrates all four pathways, and is expressed as follows:

(9) $$q_{e}\;={\;q}_{phys}+{\;q}_{chem,C=O}\;+\;q_{chem,C–O}\;+{\;q}_{\pi }$$

where the dominant contribution is physisorption ($q_{phys}$), consistent with the exothermic but low-enthalpy thermodynamic profile (Δ*H°* = −9.42 kJ mol^−1^) and the facile thermal regeneration at 393 K is confirmed across six adsorption–desorption cycles.

The KOH activation reaction pathways (supporting chemistry), the chemical reactions occurring during KOH microwave activation that generate the pore structure and surface functional groups of L-AC are expressed as follows:

Stage 1—Dehydration of KOH (*T* > 673 K):

(10) $$2\mathrm{KOH}\to K_{2}O\;+{\;H}_{2}O$$

Stage 2—Carbon gasification by K_2_O:

(11) $$K_{2}O\;+\;C\to 2K\;+\;\mathrm{CO}$$

Stage 3—CO_2_ formation via Boudouard reaction:

(12) $${\mathrm{CO}}_{2}+C\to 2\mathrm{CO}$$

Stage 4—Carbonate formation (pore generation):

(13) $$4\mathrm{KOH}+C\to K_{2}{\mathrm{CO}}_{3}+K_{2}O+2H_{2}$$

Stage 5—Potassium intercalation into carbon lattice (micropore creation):

(14) $$K_{2}{\mathrm{CO}}_{3}+2C\to 2K+3\mathrm{CO}$$

(15) $$K(g)\overset{\mathrm{intercalation}}{\to }K_{\left(\mathrm{between}\;\mathrm{graphene}\;\mathrm{layers}\right)}\to \mathrm{lattice}\;\mathrm{expansion}\to \mathrm{micropore}\;\mathrm{formation}$$

Stage 6—Washing to remove residual potassium compounds:

(16) $$K_{2}O+H_{2}O\to 2\mathrm{KOH}$$

(17) $$K_{2}{\mathrm{CO}}_{3}+H_{2}O\to 2\mathrm{KOH}+{\mathrm{CO}}_{2}$$

These six sequential reactions collectively explain the 165-fold increase in the BET surface area (8.2 → 1349 m^2^ g^−1^), the 78-fold increase in pore volume (0.01 → 0.78 cm^3^ g^−1^), the increase in I_D_/I_G_ (0.92 → 1.83), and the retention of C=O and C–O surface functional groups confirmed by the FTIR, all of which directly govern the CO_2_ adsorption performance of L-AC reported in Section 2.5, Section 2.6, Section 2.7 and Section 2.8.

Although CO_2_/N_2_ selectivity was not directly measured in this study, the developed L-AC is expected to preferentially adsorb CO_2_ over N_2_ because CO_2_ possesses a larger quadrupole moment and a smaller kinetic diameter (3.30 Å) than N_2_ (3.64 Å). In addition, the abundant ultra-micropores and oxygen-containing surface functional groups generated during microwave-assisted KOH activation provide favorable adsorption sites for CO_2_ through enhanced van der Waals and Lewis acid–base interactions. Nevertheless, a quantitative evaluation of CO_2_/N_2_ selectivity using mixed-gas breakthrough experiments or ideal adsorbed solution theory (IAST) analysis is beyond the scope of the present work, and will be investigated in future studies. Even though the adsorption performance was evaluated using a binary CO_2_/N_2_ gas mixture representative of post-combustion conditions, the effects of water vapor and acidic flue-gas components (e.g., SO_2_ and NOₓ) were not investigated. In addition, no control adsorbents prepared by conventional tube-furnace activation or biomass precursors were included for direct experimental comparison. Nevertheless, a comparison with the representative literature demonstrates that the microwave-assisted KOH activation strategy produced L-AC with competitive textural properties and CO_2_ adsorption performance. Future work will focus on multi-component breakthrough experiments under humid flue-gas conditions and direct comparisons with conventionally activated lignite- and biomass-derived activated carbons prepared under identical conditions to further evaluate CO_2_ selectivity, impurity tolerance, and the advantages of microwave-assisted activation.

### 2.8. Molecular-Level Understanding of CO_2_ Adsorption on L-AC

In order to gain additional insight regarding the mechanism of molecular-level interactions of CO_2_ adsorption onto the L-AC structure, it is possible to consider the role of micropore confinement effects, interactions between π–electrons of graphene domains and CO_2_ molecules, oxygen-containing functional groups present in the sample, and the defect-rich carbon surface. Microwaves used in the activation procedure converted low-rank lignite into porous defect-rich activated carbon, as indicated by a high surface area of 1349 m^2^ g^−1^, total pore volume of 0.78 cm^3^ g^−1^, and I_D_/I_G_ value of 1.83 obtained by Raman spectroscopy. All these characteristics create ideal conditions for molecular-level adsorption and enhance molecular-level interactions within the porous carbon material. On the molecular level, the adsorption of CO_2_ molecules on the surface of L-AC should proceed according to a reversible process of physisorption rather than chemisorption. CO_2_ is an asymmetric molecule with a pronounced quadrupole moment; hence, it will undergo favorable interactions with the graphitic π-domain through quadrupole–π interactions and dispersion interactions. The defect-rich graphitic domain created during the activation process leads to a localized electronic density distribution and thus contributes to the strength of the interactions between CO_2_ molecules and aromatic carbon planes. Such an interaction is strong enough to promote CO_2_ adsorption at low temperatures and relatively weak to enable efficient desorption during temperature-swing adsorption.

The FTIR data also suggest that oxygen-containing functional groups participate in the process of CO_2_ interaction with the surface of L-AC. The presence of C=O and C–O–C bonds means that carbonyl and ether oxygen atoms become sites for electron localization on the carbon matrix. Due to the fact that CO_2_ contains a carbon atom that is characterized by deficiency of electrons, a Lewis acid–base interaction is also possible. As was mentioned earlier, such an interaction involves no formation of a permanent chemical bond; rather, it stabilizes the molecule through electrostatic and dipole-induced interactions. Hence, oxygen-containing functional groups increase affinity towards CO_2_ molecules.

Micropore confinement effects are the other molecular-level factors contributing to efficient CO_2_ capture. According to the data obtained, it can be assumed that after the activation process numerous micropores and mesopores have been formed in the structure of L-AC. Within the micropores, the adsorption potential fields created by opposite pore walls overlap, leading to the creation of a stronger adsorption field for CO_2_ molecules. Mesopores and macropores act as channels for the diffusion of CO_2_ towards the internal porous network. The three-stage Weber–Morris intra-particle diffusion behavior suggests the role of diffusion through the meso- and macroporous structure to reach micropores filled with adsorbed CO_2_.

**Figure 12:**
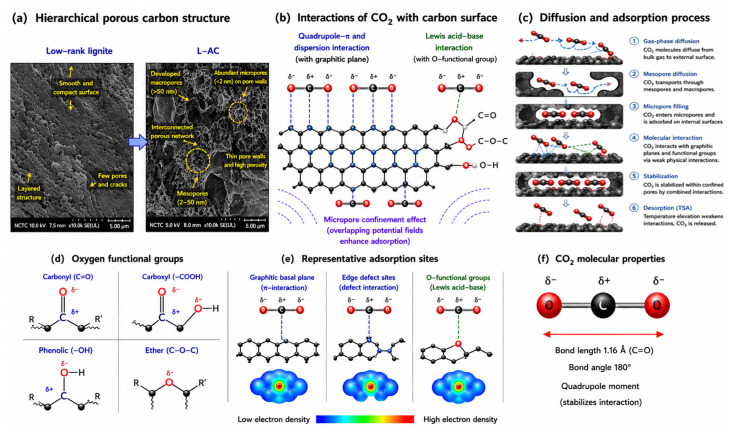
Proposed molecular-level adsorption mechanism of CO_2_ on L-AC: (**a**) hierarchical pore structure; (**b**) molecular interaction forces; (**c**) sequential adsorption process; (**d**) oxygen-containing functional groups; (**e**) adsorption sites and electron-density distribution; and (**f**) quadrupole characteristics of the CO_2_ molecule (Image generated by ChatGPT and Claude AI, 2026).

The kinetic analysis also supports the idea of the heterogeneity of molecular-level adsorption of CO_2_. Fitting of adsorption data with the general-order and pseudo-first-order model with fractal behavior of diffusion suggests that CO_2_ adsorption does not occur uniformly on the whole surface. Rather, it occurs on multiple adsorption sites located in different parts of the carbon matrix, such as π-domain, defect-rich edges, oxygen-containing groups, and micropore walls. The increasing temperature leading to decreasing adsorption capacity and negative ΔH° value prove that CO_2_ adsorption is exothermic and reversible. Considering all these factors, as shown in Figure 12, it is possible to describe the molecular-level adsorption of CO_2_ on L-AC as follows: (i) CO_2_ diffusion into adsorbent matrix; (ii) CO_2_ transport via mesoporous network; (iii) CO_2_ accumulation within microporous network; (iv) Interaction between quadrupole moment of CO_2_ and graphitic domains; (v) Weak Lewis acid–base interaction with oxygen-containing groups; and (vi) Stabilization of CO_2_ molecules within micropores.

### 2.9. Thermodynamic Adsorption Analysis

**Figure 13:**
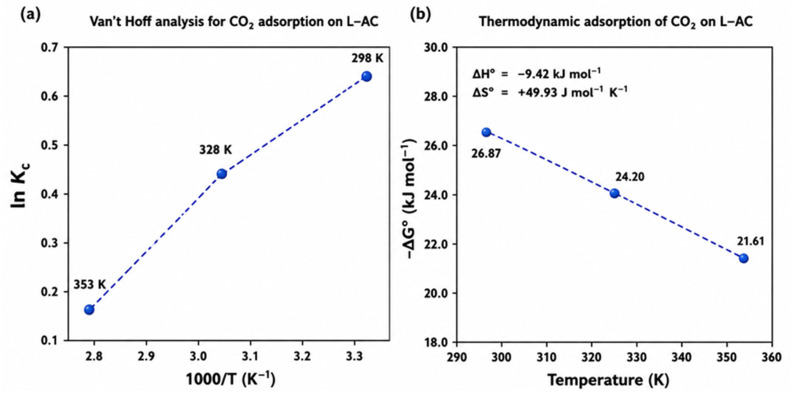
Thermodynamic adsorption behavior of CO_2_ on L-AC: (**a**) Van’t Hoff plot for Δ*H°* and Δ*S°* determination and (**b**) temperature-dependent Gibbs free energy variation. Negative Δ*G°*, negative Δ*H°*, and positive Δ*S°* confirm spontaneous, exothermic, and entropy-assisted CO_2_ physisorption on the hierarchical porous carbon structure.

The thermodynamic parameters for CO_2_ adsorption onto L-AC are summarized in Table 7 and Figure 13. Negative Δ*G*° values at all temperatures (−24.20, −26.06, and −26.87 kJ mol^−1^ at 298, 328, and 353 K, respectively) confirm that adsorption is thermodynamically spontaneous across the entire experimental range. The negative trend of Δ*G°* as the temperature increases is thermodynamically justified: although the equilibrium capacity decreases (*q_e_* = 47.34 to 21.34 mg g^−1^), the positive contribution of entropy increases (*T*Δ*S°*) and leads to the more negative values of Δ*G°*, which is typical for the physical adsorption of gases (physisorption) on microporous carbonaceous materials. The negative value of Δ*H°* (−9.42 kJ mol^−1^) confirms an exothermic process, corresponding to a temperature-related decrease in qe (Section 2.5) and breakthrough characteristics (Section 2.6). The magnitude of Δ*H°* < 40 kJ mol^−1^ justifies a physical process driven by van der Waals interactions, considering complete regenerability of the material after six cycles of loading/unloading (Section 2.6). The positive Δ*S°* (+49.93 J mol^−1^ K^−1^) shows enhanced randomness due to spatial distribution of the CO_2_ molecules between different micropores within the L-AC structure; both Δ*H°* and *T*Δ*S°* contribute to negative Δ*G°* values over the entire range of temperatures considered. The activation energy *E_a_* = 9.11 kJ mol^−1^ (*R^2^* = 0.94) calculated using the Arrhenius plot of *k_r_* falls into the range of 5–15 kJ mol^−1^, characteristic for diffusion-controlled physisorption. This result is consistent with the diffusion within mesopores found to be the main rate-controlling step (Section 2.5) using the Weber–Morris approach. Collectively, the thermodynamic profile spontaneous (Δ*G*° < 0), exothermic (Δ*H*° < 0), entropy-assisted (Δ*S*° > 0), and low-barrier (*E_a_* < 10 kJ mol^−1^) confirms physisorptive CO_2_ uptake on L-AC that is well-suited to a temperature-swing adsorption (TSA) operation with facile regeneration at 393 K. It should be noted that the present study focused on dynamic fixed-bed breakthrough adsorption to evaluate the practical CO_2_ capture performance of L-AC under post-combustion conditions. Although equilibrium adsorption isotherms (e.g., Langmuir, Freundlich, Toth, and Sips models) were not investigated, such analyses would provide complementary information on adsorption equilibrium, surface heterogeneity, and adsorption-site distribution. These equilibrium studies will be performed in future work to further establish the adsorption characteristics of L-AC over a wider range of CO_2_ pressures and temperatures.

### 2.10. Reusability and Cyclic Stability of L-AC

**Figure 14:**
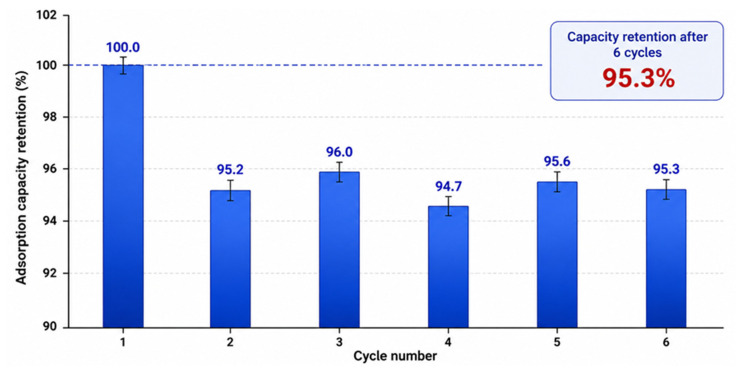
CO_2_ adsorption capacity of L-AC during six consecutive adsorption–desorption cycles. The adsorbent exhibited an excellent regeneration performance with capacity retention greater than 95%, indicating the preservation of pore structure and active adsorption sites throughout repeated thermal regeneration.

The cyclic reusability and stability of L-AC through repeated adsorption–desorption processes were further explored by investigating six adsorption–desorption cycles, with the results of which showing adsorption capacity retention in the range from 94.7% to 96.0%, as demonstrated in Figure 14. The capacity retention of the L-AC adsorbent in each adsorption–desorption cycle showed an almost identical level of cyclic stability, with a slight drop in adsorption capacity retention observed in the first adsorption–desorption cycle (i.e., from 95.8% to 95.2%), after which it continued to remain almost unchanged at 95.7%. As indicated above, the maximum adsorption capacity retention (i.e., 96.0%) was achieved in the third cycle, while the minimum one (i.e., 94.7%) was found to be in the fourth cycle.

After undergoing six adsorption–desorption cycles, L-AC could retain its initial adsorption capacity up to 95.3%, showing good adsorbent regeneration ability and excellent cyclic stability. This could be attributed to the preservation of the hierarchical porous structure and active sites of the material in repeated adsorption–desorption processes due to the relatively high regeneration temperature (i.e., 393 K). As a result, there would be no structural damage or irreversible blocking of active sites caused by pore collapse during the adsorption and desorption of CO_2_ molecules. The excellent retention of more than 95% of the initial CO_2_ adsorption capacity after six consecutive adsorption–desorption cycles demonstrates the good regeneration stability of L-AC and suggests promising long-term operational durability under the investigated conditions.

In view of the excellent stability mentioned above, this cyclic stability of the L-AC adsorbent can be well explained by considering that the adsorption process is mainly controlled by physisorption. The interactions between CO_2_ and the L-AC adsorbent at the molecular level are reversible interactions, such as quadrupole–π interactions between the CO_2_ molecules and the graphitic carbon layer; Lewis acid–base interactions with oxygen-containing surface functional groups; and the confinement effect in micropores. Because these interactions are reversible and weak, CO_2_ molecules will be easily stripped out from the microporous carbon layer during regeneration processes.

## 3. Materials and Methods

Low-rank lignite, employed as the carbonaceous precursor, was sourced directly from the Mae Moh power plant, Lampang Province, Thailand. Prior to use, the as-received lignite was mechanically ground and classified by dry sieving to obtain a uniform particle size fraction of 0.5–1.3 mm. The sized material was subsequently washed with deionized water to eliminate surface-bound clay minerals and particulate impurities, then dried overnight in a forced-air oven at 383 K until a constant mass was achieved.

Potassium hydroxide (KOH; purity ≥ 85%, ACI Labscan, Bangkok, Thailand) was used as the chemical activating agent without further purification. High-purity carbon dioxide (CO_2_; 99.9999 vol%, Linde, Bangkok, Thailand) was used as the adsorbate gas for all adsorption–desorption performance evaluations. Nitrogen gas (N_2_; 99.999 vol%, Linde, Bangkok, Thailand) was employed as the inert carrier and purge gas throughout the activation and adsorption experiments.

### 3.1. Microwave-Assisted Preparation of Low-Rank Lignite-Derived Activated Carbon

**Figure 15:**
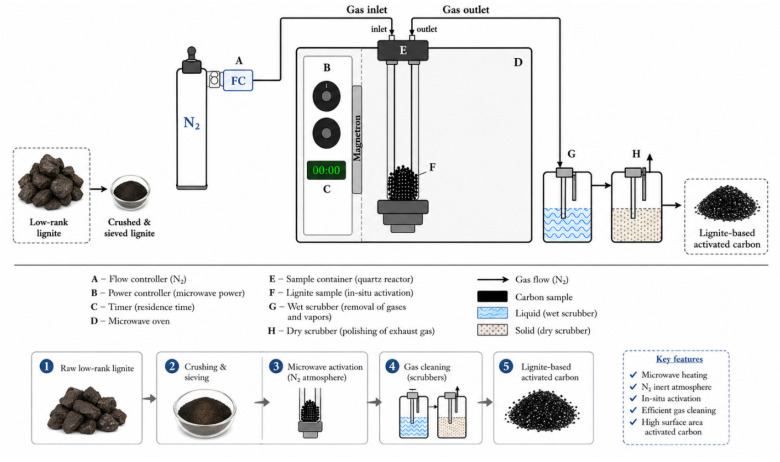
Schematic diagram of low-rank lignite-based activated carbon preparation (Image generated by ChatGPT and Claude AI, 2026).

Low-rank lignite-derived activated carbon was synthesized via KOH chemical activation under microwave-assisted heating using a modified domestic microwave oven (Samsung ME711K, Bangkok, Thialand) operating at a magnetron frequency of 2.45 GHz with a maximum output power of 800 W. The instrument was configured with an externally adjustable power controller and programmable timer to enable precise regulation of activation conditions. Before the activation process, 20 g of pretreated low-grade lignite was thoroughly mixed with KOH using different impregnation ratios and placed inside a quartz tube, which was inside the microwave chamber (Figure 15). In order to avoid oxidation that may cause damage to the carbon precursor during heating, the experiment was carried out using a steady flow of inert nitrogen (N_2_) gas at 100 mL min^−1^. After activation, the produced material was allowed to cool down to room temperature using N_2_ gas and later washed with distilled water to remove the remaining KOH and other soluble compounds, ensuring that the filtration attains neutral pH. The activated carbon synthesized under the identified optimal conditions was designated as L-AC and employed for subsequent physicochemical characterization and CO_2_ adsorption evaluation.

The activation conditions employed in this study were selected based on preliminary optimization and the previous literature to obtain a representative high-performance activated carbon suitable for investigating CO_2_ adsorption behavior. The primary objective of this work was to evaluate the adsorption performance, kinetics, thermodynamics, and adsorption mechanism of the prepared L-AC rather than to systematically optimize the activation parameters. Future studies will systematically investigate the effects of the KOH impregnation ratio, microwave power, irradiation time, and activation temperature to establish quantitative structure–property relationships governing pore development and CO_2_ adsorption performance.

### 3.2. Physicochemical Characterization of Activated Carbon

Textural properties of the raw low-rank lignite and L-AC were evaluated by N_2_ adsorption–desorption analysis at 77 K (Quantachrome Autosorb-1, Anton Paar QuantaTec, Boynton Beach, FL, USA); specific surface area, pore size distribution, and total pore volume were determined by the BET, BJH, and single-point methods, respectively. Surface morphology was examined by scanning electron microscopy (SEM; Hitachi S–3400N, high vacuum, Hitachi, Tokyo, Japan). Structural order and degree of graphitization were assessed by Raman spectroscopy (WITec alpha300 R, WITec GmbH, Ulm, Germany; 532 nm laser, 5 mW, 100× objective; 800–2000 cm^−1^); the D-band (~1348 cm^−1^) to G-band (~1581 cm^−1^) intensity ratio (I_D_/I_G_) was determined by Lorentzian peak fitting as a quantitative index of structural defect density [23,27]. The elemental composition, specifically the carbon (C), hydrogen (H), nitrogen (N), and sulfur (S) contents, was quantified by ultimate analysis using a LECO TruSpec 628 CHNS analyzer (LECO Corporation, St. Joseph, MI, USA); the oxygen (O) content was estimated by difference from the remaining mass fraction. Surface functional groups were identified by Fourier-transform infrared (FTIR) spectroscopy (PerkinElmer FTIR Spectrometer, ATR, PerkinElmer, High Wycombe, UK) over the wavenumber range of 4000–400 cm^−1^, with spectra acquired in transmission mode using the KBr pellet technique.

### 3.3. Apply to CO_2_ Adsorption: Fixed-Bed Adsorption Test

**Figure 16:**
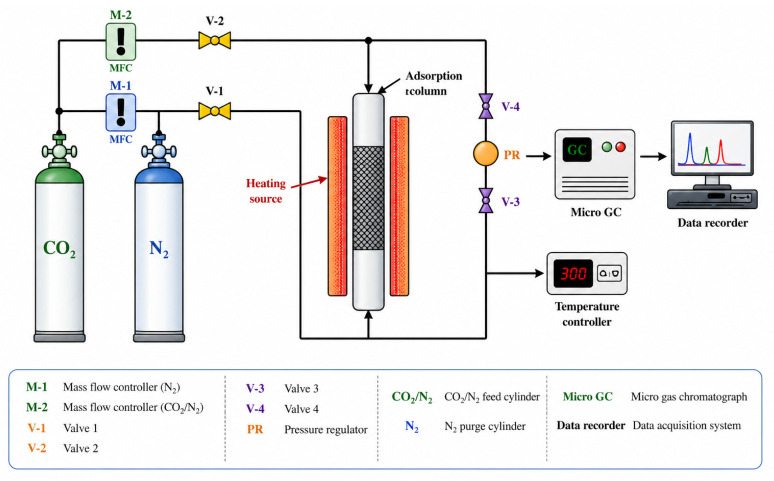
Schematic diagram of the fixed bed adsorption–desorption setup (Image generated by ChatGPT and Claude AI, 2026).

The CO_2_ capture performance of L-AC was assessed using a laboratory–scale fixed-bed adsorption system consisting of a vertically oriented stainless-steel column (total length: 400 mm; outer diameter: 10 mm; inner diameter: 6 mm) connected in-line to a Micro GC analyzer (Agilent 490–GC, Agilent Technologies, Wellington, New Zealand) for continuous real-time quantification of effluent gas composition (Figure 16). A precisely weighed mass of 1.0 g of the adsorbent was uniformly packed into the column for each experimental run. Each adsorption–desorption cycle comprised three sequential stages: pre-treatment, adsorption, and thermal regeneration. Prior to each adsorption run, the packed bed was thermally conditioned by ramping from ambient temperature to 383 K under a continuous flow of high-purity N_2_ (99.999 vol%) at 50 mL min^−1^ for 30 min. This pre-treatment process was effective for the thorough removal of moisture and other pre-adsorbed materials from the adsorbent surface to create reproducible initial conditions for each cycle. Following that, a feed gas comprising 15 vol% CO_2_ mixed with N_2_ was flowed upwards through the bed at a volumetric flow rate of 5 mL min^−1^. The adsorption tests were performed isothermally at three different operating temperatures of 298, 328, and 353 K at 1 atm pressure until breakthrough was observed when the concentration of CO_2_ in the outlet gas stream reached the inlet feed concentration. Afterward, thermal regeneration was started by changing the inlet gas feed from CO_2_/N_2_ to N_2_ at a higher temperature of 393 K and maintaining the same volumetric flow rate of 5 mL min^−1^. This process led to full desorption of CO_2_ and activated the adsorbent for the next cycle. To examine the performance of L-AC regarding cyclic stability and reusability during its practical application, a series of six successive adsorption–desorption cycles was conducted. The amount of CO_2_ adsorption was measured from the breakthrough curve through numerical calculation of the area under the curve. The equilibrium CO_2_ adsorption capacity, *q_e_* (mg g^−1^), can be calculated from the breakthrough curve using Equation (18):

(18) $$q_{e}=\frac{Q\times \int_{0}^{t_{eq}}(1-C/C_{0})dt}{m}\times \frac{1}{24.465}\times 44.01$$

In this equation, Q refers to the volumetric flow rate of the feed gas (mL min^−1^), *C*/*C*_0_ refers to the dimensionless ratio of outlet to inlet CO_2_ concentration, *t_eq_* is the time needed to achieve adsorption equilibrium (min), and m is the mass of the adsorbent in the column (g). The integral accounts for the area above the breakthrough curve (min) and can be used to measure the total equivalent time of CO_2_ adsorption; the value of the area is numerically integrated using the experimental data of the breakthrough curve. Multiplying the Q parameter by the integral gives the total volume of CO_2_ captured (mL). The calculated volume is then converted into a number of moles by dividing it with the molar volume of an ideal gas at 298 K and 1 atm (24.465 mL mmol^−1^, calculated based on *RT*/*P* = 8.314 × 298 / 101,325 = 0.02446 m^3^ mol^−1^). The value thus obtained is then multiplied by the molecular weight of CO_2_ (44.01 mg mmol^−^1, equal to 12.01 + 2 × 16.00 g mol^−1^) to calculate the amount of CO_2_ captured (mg). Dividing the result by the mass of the adsorbent (m) gives the value of qe in mg g^−1^. This formula ensures dimensional homogeneity, since the units cancel each other out: mL min^−1^ × min × mmol mL^−1^ × mg mmol^−1^ × g^−1^ = mg g^−1^. It is important to note that the molar volume of gas used in the calculations is strictly applicable only to 298 K; hence, for experiments performed at 328 and 353 K, corrected values of 26.840 and 28.898 mL mmol^−1^, respectively, should be used.

### 3.4. Kinetic Adsorption

The CO_2_ adsorption kinetics of L-AC were investigated by fitting experimental uptake data (*q_t_*, mg g^−1^) as a function of contact time (*t*, min) at 298, 328, and 353 K to seven kinetic models: pseudo-first-order (PFO), pseudo–second–order (PSO), Elovich, Avrami fractional-order, fractal-like pseudo-first-order (FL-PFO), fractal–like pseudo–second–order (FL-PSO), and general-order. The PFO and PSO models describe adsorption governed by available surface sites and chemisorptive electron exchange, respectively. The Elovich model characterizes adsorption on energetically heterogeneous surfaces via the initial adsorption rate α (mg g^−1^ min^−1^) and desorption constant β (g mg^−1^). The Avrami model extends PFO by incorporating a fractional exponent *n*AV to capture multi-stage kinetics, while the FL-PFO and FL-PSO models introduce a fractal temporal exponent α (0 < α ≤ 1) to account for time-dependent rate constants on heterogeneous surfaces. The general-order model allows the reaction order *n* to vary freely, providing the most flexible mechanistic description. All parameters were determined by nonlinear regression analysis using the Levenberg–Marquardt technique. The goodness of fit for the models was analyzed using the coefficient of determination (*R*^2^), sum of squares of errors (SSE), and the chi-squared value (*χ*^2^). The rate-limiting step was examined using the Weber–Morris intra-particle diffusion (IPD) equation, which is given as follows: *q_t_* = *k_id_t*^0.5^ +C. Here, *k_id_* (mg g^−1^ min^−0^·^5^) stands for the diffusion rate constant and C (mg g^−1^) is the boundary layer thickness. Linearity and intercept properties of *q_t_* versus *t*^0.5^ were used to identify whether it followed the one-stage intra-particle diffusion process or multiple steps with external film diffusion, mesopore diffusion, and micropore saturation.

### 3.5. Thermodynamic Adsorption

The thermodynamic parameters of CO_2_ adsorption onto L-AC were evaluated at 298, 328, and 353 K using the van’t Hoff approach. The dimensionless equilibrium constant *K_c_* was calculated as follows:

(19) $$K_{c}\;=\;\frac{q_{e}\cdot \mathrm{RT}}{P_{CO_{2}}/P_{0}}$$

where *q*e is the equilibrium adsorption capacity (mol kg^−1^), *R* = 8.314 J mol^−1^ K^−1^, *T* is absolute temperature (K), *P_CO_*_2_ = 15.20 kPa (partial pressure at 15 vol% and 1 atm), and *P*_0_ = 100 kPa (standard pressure). The standard Gibbs free energy (Δ*G*°), enthalpy (Δ*H*°), and entropy (Δ*S*°) were obtained from the following equations:

(20) $$\Delta G{}^{\circ}\;=-\mathrm{RTln}K_{c}$$

(21) $$\ln K_{c}=\frac{\Delta S{}^{\circ}}{R}-\frac{\Delta H{}^{\circ}}{\mathrm{RT}}$$

with Δ*H*° and Δ*S*° derived from the slope and intercept of the linear van’t Hoff plot of ln *K_c_* versus 1/*T* (Figure 11). The activation energy (*E_a_*) was estimated from the Arrhenius equation applied to the general-order rate constant *k_r_*:

(22) $$\ln k_{r}\;=\;\mathrm{lnA}-\frac{E_{a}}{\mathrm{RT}}$$

## 4. Conclusions

This study is concerned with upgrading low-ranked lignite coal from Mae Moh to produce high-quality activated carbon (L-AC) using microwave-assisted KOH activation for carbon dioxide removal after combustion. Activation resulted in a significant increase in the BET surface area (1349 m^2^ g^−1^g compared to 8.2 m^2^ g^−1^) and in total pore volume (0.78 cm^3^ g^−1^ vs. 0.01 cm^3^ g^−1^). As such, this process afforded the formation of a developed hierarchical structure of micropores and mesopores. The formation of this microstructure was confirmed by scanning electron microscopy, N_2_ adsorption analysis, and Raman spectroscopy (I_D_/I_G_ = 1.83). Fourier-transform infrared spectroscopy confirmed the preservation of surface C=O and C–O–C functional groups that were expected to improve CO_2_ adsorption. Fixed-bed breakthrough experiments (15 vol% CO_2_, 1 atm; 298–353 K) yielded equilibrium CO_2_ uptakes of 47.34, 34.37, and 21.34 mg g^−1^, respectively, consistent with exothermic physisorption confirmed thermodynamically by Δ*H°* = −9.42 kJ mol^−1^, Δ*G°* < 0 at all temperatures, and *Eₐ* = 9.11 kJ mol^−1^. The Thomas and Yoon–Nelson breakthrough curve models effectively modeled the data, showing correlation coefficients within the range of 0.9996–0.9998. L-AC proved to be stable through six repeated adsorption–desorption cycles performed at 393 K and thus is suitable for applications in temperature swing adsorption techniques. The amount of CO_2_ sorption achieved (1.07 mmol g^−1^) by L-AC at 328 K was higher than that of the AC derived from anthracite (0.91 mmol g^−1^) and significantly higher than that of an AC/MXene composite (0.03 mmol g^−1^). Among seven investigated models, the general-order and FL-PFO models gave the highest statistics, with the decrease in the reaction order (*n* = 1.82–1.52) and fractal dimension (*α* = 0.78–0.70) indicating that the effect of temperature on surface heterogeneity became weaker. According to the Weber–Morris model, the transport mechanism of CO_2_ adsorption by L-AC consisted of three steps: Stage I included rapid external film diffusion; Stage II corresponded to the rate limiting process of mesopore diffusion (*k_id2_* = 0.31–5.35 mg g^−1^ min^−0^·^5^); and the last stage, Stage III, involved micropore saturation. These findings clearly indicate that L-AC can be considered a cheap and reusable carbonaceous material prepared from a readily available low-grade coal. Future work should address CO_2_/N_2_ selectivity under mixed-gas and humid conditions, pilot-scale fixed-bed performance, and the potential benefit of nitrogen doping to further enhance adsorption capacity and selectivity, with the aim of advancing this material toward deployment in post-combustion CO_2_ capture systems. Future work will systematically optimize the activation parameters, including the KOH impregnation ratio, microwave power, irradiation time, and activation temperature, to establish the quantitative structure–property relationships governing pore development and CO_2_ adsorption performance. Equilibrium adsorption isotherm measurements and multi-component breakthrough experiments under realistic flue-gas conditions will also be conducted to provide a more comprehensive evaluation of the adsorption behavior and practical applicability of L-AC. Given the abundance and low cost of Mae Moh lignite, together with the rapid and efficient microwave-assisted activation process, L-AC exhibits promising potential for large-scale production. Further studies will include techno-economic assessments, pilot-scale validation, and integration with practical post-combustion CCUS systems to evaluate its industrial feasibility and long-term commercial potential.

## Figures and Tables

**Table 1:** Surface area, pore volume, average pore size, and ultimate analysis of low-rank lignite and L-AC.

Sample	Surface Area (m^2^ g^−1^)	Pore Volume (cm^3^ g^−1^)	Avg. Pore Size (nm)	Pore Types	Ultimate Analysis (wt.%)			
					C	H	N	S
Low-rank lignite	8.2	0.01	89.82	Macro	47.89	3.67	1.94	2.52
L-AC	1349	0.78	5.61	Meso	54.56	1.62	1.62	0.60

**Table 2:** Comparison of physicochemical properties and CO_2_ adsorption performance of representative activated carbons.

Adsorbent	Precursor	BET Surface Area (m^2^ g^−1^)	CO_2_ Adsorption Capacity	Adsorption Conditions	Reference
L-AC (This work)	Low-rank lignite	1349	47.34 mg g^−1^ (1.08 mmol g^−1^)	15 vol% CO_2_/N_2_, 298 K, fixed-bed	This work
Coal-derived activated carbon	Coal	661–1100	0.03–0.91 mmol g^−1^	Post-combustion CO_2_	Creamer and Gao [32]
Pine nut shell activated carbon	Pine nut shell	1000–1400	3.5–4.3 mmol g^−1^	Pure CO_2_, 298 K	Deng et al. [33]
Rice husk activated carbon	Rice husk	1500–3228	4.11–4.18 mmol g^−1^	Pure CO_2_, 298 K	Li and Xiao [34]
Palm shell activated carbon	Palm shell	1090–1210	1.0–1.6 mmol g^−1^	Pure CO_2_, 298 K	Ello et al. [35]
Coconut shell activated carbon	Coconut shell	900–1300	1.3–2.0 mmol g^−1^	Pure CO_2_, 298 K	Huang et al. [36]

**Table 3:** Thomas and Yoon–Nelson model parameters for CO_2_ adsorption on L-AC.

Model	Parameter	Unit	298 K	328 K	353 K
Thomas	*k_Th_*	mL mg^−1^ min^−1^	0.9075	1.3318	2.5799
	*q*_0_	mg g^−1^	135	90	50
	R^2^	—	0.9998	0.9997	0.9996
Yoon–Nelson	*k_YN_*	min^−1^	0.245	0.3267	0.588
	*τ*_50_	min	27	18	10
Bed performance	*t_b_*	min	15	9	5
	*t_s_*	min	39	27	15
	MTZ	mm	246.2	266.7	266.7
	BUE	%	34.9	38.5	43.2

*k_Th_* = Thomas rate constant; *q*_0_ = Thomas adsorption capacity; *k_YN_* = Yoon–Nelson rate constant; *τ*_50_ = time for 50% adsorbent breakthrough; *t_b_* = breakthrough time (*C*/*C*_0_ = 0.05); *t_s_* = saturation time; MTZ = mass transfer zone length; BUE = bed utilization efficiency = *q*_e,exp_/*q*_0_ × 100%.

**Table 4:** CO_2_ adsorption kinetics model for the L-AC adsorbent.

Model	Parameter	298 K	328 K	353 K
PFO [37] $q_{t}=q_{e}\left[1-\exp\left(-k_{1}\cdot t\right)\right]$	*q_e_* (mg g^−1^)	47.34	34.37	21.34
	*k_1_* (min^−1^)	0.0895	0.0985	0.1038
	*R^2^*	0.9995	0.9990	0.9987
	*SSE*	1.616	1.588	0.765
	*χ^2^*	0.118	0.196	0.112
PSO [38] $q_{t}=\frac{k_{2}\cdot q_{e}^{2}\cdot t}{1+q_{e}\cdot k_{2}\cdot t}$	*q_e_* (mg g^−1^)	55.08	39.51	24.40
	*k_2_*	1.949 × 10^−3^	3.107 × 10^−3^	5.381 × 10^−3^
	*R^2^*	0.9900	0.9944	0.9948
	*SSE*	31.021	8.900	3.154
	*χ^2^*	1.005	0.400	0.238
Elovich [39] $q_{t}=\frac{1}{\beta }\cdot \ln\left(1+\alpha \cdot \beta \cdot t\right)$	*α* (mg g^−1^ min^−1^)	10.83	9.69	6.65
	*β* (g mg^−1^)	0.0831	0.1210	0.1999
	*R^2^*	0.9634	0.9690	0.9681
	*SSE*	113.710	49.291	19.365
	*χ^2^*	3.885	2.326	1.487
Avrami [40] $q_{t}=q_{e}\times \left[1-\exp{\left(-(k_{av}\times t)\right)}^{n_{av}}\right]$	*q_e_* (mg g^−1^)	47.34	34.65	21.53
	*k_av_* (min^−1^)	0.0895	0.0969	0.1020
	*n_av_*	1.00	0.9240	0.9149
	*R^2^*	0.9995	0.9998	0.9997
	*SSE*	1.616	0.379	0.188
	*χ^2^*	0.121	0.034	0.014
FL-PFO [41] $q_{t}=q_{e}\cdot \left(1-\exp\left(-k_{1}\cdot t^{\alpha }\right)\right)$	q_e_ (mg g^−1^)	47.34	34.65	21.53
	k_1_	0.0892	0.1158	0.1239
	α	1.00	0.9240	0.9149
	*R^2^*	0.9995	0.9998	0.9997
	*SSE*	1.616	0.379	0.188
	*χ^2^*	0.121	0.034	0.014
FL-PSO [41] $q_{t}=\frac{k_{2}\cdot q_{e}^{2}\cdot t^{\alpha }}{1+k_{2}\cdot q_{e}\cdot t^{\alpha }}$	q_e_ (mg g^−1^)	50.85	37.03	22.92
	k_2_	1.290 × 10^−3^	2.260 × 10^−3^	3.925 × 10^−3^
	α	1.30	1.25	1.25
	*R^2^*	0.9960	0.9979	0.9984
	*SSE*	12.391	3.355	0.987
	*χ^2^*	0.603	0.278	0.125
General order [42] $q_{t}=q_{e}-\frac{q_{e}}{{\left[t\cdot k_{r}\cdot q_{e}^{n-1}\cdot \left(n-1\right)+1\right]}^{1/(n-1)}}$	q_e_ (mg g^−1^)	47.35	34.87	21.72
	kᵣ [h^−1^ (g mg^−1^)^n–1^]	0.0892	0.0602	0.0616
	n	1.00	1.16	1.20
	*R^2^*	0.9995	0.9996	0.9997
	*SSE*	1.626	0.655	0.207
	*χ^2^*	0.118	0.083	0.030

**Table 5:** Intra-particle diffusion models parameters for the CO_2_ adsorption onto L-AC.

Temp.	Stage	Diffusion Region	*k_id_* (mg g^−1^ min^−0.5^)	C (mg g^−1^)	*R* ^2^	Fitting Range (t^0.5^, min^0.5^)
298 K	Stage I	Film diffusion	7.52	5.41	0.9853	1.0–2.8
	Stage II	Intra-particle diffusion	7.84	8.88	0.9987	3.0–7.0
	Stage III	Equilibrium	0.52	46.3	0.9621	7.0–9.0
328 K	Stage I	Film diffusion	5.42	4.32	0.9817	1.0–2.8
	Stage II	Intra-particle diffusion	6.08	6.76	0.9984	3.0–7.0
	Stage III	Equilibrium	0.34	33.88	0.9389	7.0–9.0
353 K	Stage I	Film diffusion	3.68	3.18	0.9786	1.0–2.8
	Stage II	Intra-particle diffusion	4.41	4.9	0.9971	3.0–7.0
	Stage III	Equilibrium	0.19	20.77	0.9045	7.0–9.0

**Table 6:** Comparison of Weber–Morris IPD parameters for CO_2_ adsorption on carbonaceous adsorbents.

Material (Precursor)	*T* (K)	*q_e_* (mg g^−1^)	*k_id1_* (mg g^−1^ min^−0.5^)	*k_id2_* (mg g^−1^ min^−0.5^)	*k_id_*_3_ (mg g^−1^ min^−0.5^)	Rate-Limiting Stage	Best Kinetic Model
L-AC (Lignite, KOH) This work	298	47.34	7.52	7.84	0.52	Stage II (mesopore diffusion)	General-order/FL-PFO
	328	34.37	5.42	6.08	0.34		
	353	21.34	3.68	4.41	0.19		
AC (Olive waste, KOH) [43]	298	40.04	7.20	2.15	0.19	Stage II	Avrami
	308	35.20	9.80	1.84	0.22	Stage II	Avrami
	318	28.96	11.40	1.12	0.28	Stage II	Avrami
AHC (Pine sawdust hydrochar, KHCO_3_) [44]	298	145.2	–	–	0.08–0.30	Film + IPD	Avrami
RSS–AC (Rubber seed shell, IL) [45]	298	95.04	–	–	–	Film + IPD	Avrami (fractal)
	313	86.24	–	–	–	Film + IPD	Avrami (fractal)
GAC (Anthracite, physical activation) [46]	278	–	–	4.90	–	Stage II	Avrami
	298	–	–	3.20	–	Stage II	Avrami
	318	–	–	0.40	–	Stage II	Avrami
GAC (Glycerol reforming syngas) [47]	298	–	–	–	–	Multi-stage IPD	Avrami/PSO
Malaysian coal [48]	273–318	–	–	–	–	Pore diffusion	IPD (single stage)

**Table 7:** Thermodynamic parameters for CO_2_ adsorption onto L-AC.

*T* (K)	*q_e_* (mg g^−1^)	*q_e_* (mol kg^−1^)	*K*_c_	ln *K*_c_	Δ*G*° (kJ mol^−1^)	Δ*H*° (kJ mol^−1^)	Δ*S*° (J mol^−1^ K^−1^)	*E_a_* (kJ mol^−1^)
298	47.34	1.069	17,438.27	9.7664	−24.20	−9.42	49.93	9.11
328	34.37	0.788	14,138.51	9.5567	−26.06			
353	21.34	0.491	9481.57	9.1571	−26.87			

## Data Availability

Data are contained within the article: The original contributions presented in this study are included in the article. Further inquiries can be directed to the corresponding author.

## References

[1] Yoro K.O., Daramola M.O., Rahimpour M.R., Farsi M., Makarem M.A. (2020). Chapter 1-CO_2_ emission sources, greenhouse gases, and the global warming effect. Advances in Carbon Capture.

[2] Monteagudo J.M., Durán A., Alonso M., Stoica A.-I. (2025). Investigation of effectiveness of KOH-activated olive pomace biochar for efficient direct air capture of CO_2_. Sep. Purif. Technol..

[3] Shi S., Hu Y.H. (2025). 2024, a landmark year for climate change and global carbon capture, utilization, and storage: Annual progress review. Energy Sci. Eng..

[4] Hu X., Liu L., Luo X., Xiao G., Shiko E., Zhang R., Fan X., Zhou Y., Liu Y., Zeng Z. (2020). A review of N-functionalized solid adsorbents for post-combustion CO_2_ capture. Appl. Energy.

[5] Du J., Yang W., Xu L., Bei L., Lei S., Li W., Liu H., Wang B., Sun L. (2024). Review on post-combustion CO_2_ capture by amine blended solvents and aqueous ammonia. Chem. Eng. J..

[6] Hanson E., Nwakile C., Hammed V.O. (2025). Carbon capture, utilization, and storage (CCUS) technologies: Evaluating the effectiveness of advanced CCUS solutions for reducing CO_2_ emissions. Results Surf. Interfaces.

[7] Zentou H., Hoque B., Abdalla M.A., Saber A.F., Abdelaziz O.Y., Aliyu M., Alkhedhair A.M., Alabduly A.J., Abdelnaby M.M. (2025). Recent advances and challenges in solid sorbents for CO_2_ capture. Carbon Capture Sci. Technol..

[8] Mouctar M.H., Hassan M.G., Bimbo N., Abbas S.Z., Shigidi I. (2025). Comparative assessment and deployment of zeolites, mofs, and activated carbons for CO_2_ capture and geological sequestration applications. Inventions.

[9] Kreetachat T., Imman S., Suriyachai N., Kreetachat N., Hongthong S., Suwannahong K., Phadee P., Janthakhot A., Wongcharee S. (2026). Multi-index evaluation of surface water quality at the transboundary section of the mekong river: A case study of nakhon phanom province, Thailand. Environ. Chall..

[10] Wongcharee S., Suriyachai N., Kreetachat T., Nukunudompanich M., Jadsadajerm S., Imman S. (2026). Synergistic Ni–Cu/char bimetallic catalysts for enhanced hydrogen production from corn stover bio-oil via steam reforming. RSC Adv..

[11] Xuan K., Zhong L., Othman R.M., Lithoxoos G.P., Almansour F., Shakhs A.N., Liu Y., Zhu X., Duan N., Sun X. (2025). On CO_2_ capture capacity and mechanisms for zeolite templated carbon, MOF-199, and 13X zeolite in dry and humid conditions. Sep. Purif. Technol..

[12] Zhang R., Xie Z., Ge Q., Zhu X. (2024). Recent advancements in integrating CO_2_ capture from flue gas and ambient air with thermal catalytic conversion for efficient CO_2_ utilization. J. CO_2_ Util..

[13] Wongcharee S., Kandasamy B., Govindasamy P., Tansomros P., Hongthong S., Sangsida W., Phibanchon S., Chotigawin R., Pahasup-anan T., Pannaracha P. (2025). Pig bone-derived biochar from food industry waste for heavy metal remediation: Sustainable consumption and production. Results Eng..

[14] Kreetachat T., Imman S., Suriyachai N., Khaowdang S., Chanthakhot A., Janthakhot A., Wongcharee S., Sangsida W., Hongthong S., Suwannahong K. (2026). Co-pyrolyzed sawdust–polypropylene biochar as a sustainable adsorbent for heavy-metal removal in wastewater. Appl. Water Sci..

[15] Monteagudo J.M., Durán A., Zhao Y., Monteagudo J. (2026). CO_2_ capture by olive pomace biochar: Effect of relative humidity, isosteric heat of adsorption, and a preliminary life cycle assessment investigation. Sep. Purif. Technol..

[16] Arifutzzaman A., Musa I.N., Aroua M.K., Saidur R. (2023). MXene based activated carbon novel nano-sandwich for efficient CO_2_ adsorption in fixed-bed column. J. CO_2_ Util..

[17] Rashidi N.A., Yusup S., Hameed B.H. (2013). Kinetic studies on carbon dioxide capture using lignocellulosic based activated carbon. Energy.

[18] Chen T., Liu H., Bie R. (2020). Temperature rise characteristics of coal-KOH adduct under microwave heating and the properties of resultant activated carbon for catalytic methane decomposition. J. Anal. Appl. Pyrolysis.

[19] Yan L., Sorial G.A. (2011). Chemical activation of bituminous coal for hampering oligomerization of organic contaminants. J. Hazard. Mater..

[20] Gong G.-Z., Xie Q., Zheng Y.-F., Ye S.-F., Chen Y.-F. (2009). Regulation of pore size distribution in coal-based activated carbon. New Carbon Mater..

[21] Feng Y., Meng X., Li J., Xue N., Li W., Sun M., Chen J., Wang X., Zhou R., Zhuang W. (2026). Rapid microwave synthesis of nitrogen-doped ultramicroporous coal-based carbon with enhanced CO_2_ adsorption performance. Sustain. Carbon Mater..

[22] Khalid B., Meng Q., Akram R., Cao B. (2016). Effects of KOH activation on surface area, porosity and desalination performance of coconut carbon electrodes. Desalin. Water Treat..

[23] Xu C., Li H., Lu J., Lu Y., Shi S., Ye Q., Li M., Wang Z. (2023). An investigation into the modification of microwave-assisted oxidation in the macromolecular structure of coal via XRD and raman spectroscopy. Fuel.

[24] Foong S.Y., Liew R.K., Yek P.N.Y., Han C.S., Phang X.Y., Chen X., Chong W.W.F., Verma M., Lam S.S. (2023). Microwave heating combined with activated carbon reaction bed: An energy-saving approach to convert seawater into freshwater. Energy.

[25] Jawad A.H., Mehdi Z.S., Ishak M.A.M., Ismail K. (2018). Large surface area activated carbon from low-rank coal via microwave-assisted KOH activation for methylene blue adsorption. Desalin. Water Treat..

[26] Kuloglija S., Kropik I.-M., Ahmed A.E.G., Kalman V., Windbacher A., Jordan C., Konior A., Abbaspour N., Steinacher N., Winter F. (2026). Isotherms and kinetics of CO_2_ adsorption on biochar-based activated carbon for sustainable climate solutions. Sep. Purif. Technol..

[27] Cheng N., Pan J., Shi M., Hou Q., Han Y. (2022). Using Raman spectroscopy to evaluate coal maturity: The problem. Fuel.

[28] Chen Y., Rong W., Jia Z., Dang R., Meng H., Jiang T. (2024). Preparation of Ru/C hydrogen evolution catalyst by mixed salt co-electroreduction of RuCl_3_*nH_2_O and CO_2_. Sep. Purif. Technol..

[29] Zhang W., Jiang S., Wang K., Wang L., Xu Y., Wu Z., Shao H., Wang Y., Miao M. (2015). Thermogravimetric dynamics and ftir analysis on oxidation properties of low-rank coal at low and moderate temperatures. Int. J. Coal Prep. Util..

[30] Alivand M.S., Mazaheri O., Wu Y., Stevens G.W., Scholes C.A., Mumford K.A. (2020). Preparation of nanoporous carbonaceous promoters for enhanced CO_2_ absorption in tertiary amines. Engineering.

[31] Donohue M.D., Aranovich G.L. (1998). Classification of gibbs adsorption isotherms. Adv. Colloid Interface Sci..

[32] Creamer A.E., Gao B. (2016). Carbon-based adsorbents for postcombustion CO_2_ capture: A critical review. Environ. Sci. Technol..

[33] Deng S., Wei H., Chen T., Wang B., Huang J., Yu G. (2014). Superior CO_2_ adsorption on pine nut shell-derived activated carbons and the effective micropores at different temperatures. Chem. Eng. J..

[34] Li M., Xiao R. (2019). Preparation of a dual pore structure activated carbon from rice husk char as an adsorbent for CO_2_ capture. Fuel Process. Technol..

[35] Ello A.S., de Souza L.K., Trokourey A., Jaroniec M. (2013). Development of microporous carbons for CO_2_ capture by KOH activation of African palm shells. J. CO_2_ Util..

[36] Huang P.-H., Cheng H.-H., Lin S.-H. (2015). Adsorption of carbon dioxide onto activated carbon prepared from coconut shells. J. Chem..

[37] Ezzati R. (2020). Derivation of pseudo-first-order, pseudo-second-order and modified pseudo-first-order rate equations from langmuir and freundlich isotherms for adsorption. Chem. Eng. J..

[38] Wu F.-C., Tseng R.-L., Huang S.-C., Juang R.-S. (2009). Characteristics of pseudo-second-order kinetic model for liquid-phase adsorption: A mini-review. Chem. Eng. J..

[39] Aharoni C., Tompkins F. (1970). Kinetics of adsorption and desorption and the elovich equation. Advances in Catalysis.

[40] Cardoso N.F., Lima E.C., Pinto I.S., Amavisca C.V., Royer B., Pinto R.B., Alencar W.S., Pereira S.F. (2011). Application of cupuassu shell as biosorbent for the removal of textile dyes from aqueous solution. J. Environ. Manag..

[41] Revellame E.D., Fortela D.L., Sharp W., Hernandez R., Zappi M.E. (2020). Adsorption kinetic modeling using pseudo-first order and pseudo-second order rate laws: A review. Clean. Eng. Technol..

[42] Ngô L.C., Winkler F. (2010). Rational general solutions of first order non-autonomous parametrizable ODEs. J. Symb. Comput..

[43] Jedli H., Almonnef M., Rabhi R., Mbarek M., Abdessalem J., Slimi K. (2024). Activated carbon as an adsorbent for CO_2_ capture: Adsorption, kinetics, and RSM modeling. ACS Omega.

[44] Vega M.F., Díaz-Faes E., Barriocanal C. (2024). Kinetic and mechanistic study of CO_2_ adsorption on activated hydrochars. J. CO_2_ Util..

[45] Fatima S.S., Borhan A., Ayoub M., Ghani N.A. (2023). Modeling of CO_2_ adsorption on surface-functionalized rubber-seed shell activated carbon: Isotherm and kinetic analysis. Processes.

[46] Wang B., Wang S., Yan H., Bai Y., She Y., Zhang F. (2023). Synthesis and enhanced oil recovery potential of the bio-nano-oil displacement system. ACS Omega.

[47] Maceiras R., Feijoo J., Perez-Rial L., Alvarez-Feijoo M.A., Eslami N. (2024). Influence of activated carbon granulometry on H_2_ purification in glycerol reforming syngas: Adsorption and kinetic analysis. Energies.

[48] Abunowara M., Bustam M.A., Sufian S., Babar M., Eldemerdash U., Mukhtar A., Ullah S., Assiri M.A., Al-Sehemi A.G., Lam S.S. (2023). High pressure CO_2_ adsorption onto malaysian mukah-balingian coals: Adsorption isotherms, thermodynamic and kinetic investigations. Environ. Res..

[49] Glenna D.M., Jana A., Xu Q., Wang Y., Meng Y., Yang Y., Neupane M., Wang L., Zhao H., Qian J. (2023). Carbon capture: Theoretical guidelines for activated carbon-based CO_2_ adsorption material evaluation. J. Phys. Chem. Lett..

